# Ca_v_1.3 calcium channels are full-range linear amplifiers of firing frequencies in lateral DA SN neurons

**DOI:** 10.1126/sciadv.abm4560

**Published:** 2022-06-08

**Authors:** Josef Shin, Lora Kovacheva, Dominique Thomas, Strahinja Stojanovic, Christopher J. Knowlton, Johanna Mankel, Johannes Boehm, Navid Farassat, Carlos Paladini, Jörg Striessnig, Carmen C. Canavier, Gerd Geisslinger, Jochen Roeper

**Affiliations:** 1 Goethe University, Institute of Neurophysiology, Neuroscience Center, Frankfurt am Main, Germany.; 2 Pharmazentrum Frankfurt/ZAFES, Institute of Clinical Pharmacology, Frankfurt am Main, Germany.; 3 Fraunhofer Institute for Translational Medicine and Pharmacology ITMP and Fraunhofer Cluster of Excellence for Immune Mediated Diseases CIMD, Frankfurt am Main, Germany.; 4 Department of Cell Biology and Anatomy, School of Medicine, Louisiana State University Health Sciences Center, New Orleans, LA, USA.; 5 UTSA Neuroscience Institute, University of Texas at San Antonio, San Antonio, TX, USA.; 6 University of Innsbruck, Department of Pharmacology and Toxicology, Center for Molecular Biosciences, Innsbruck, Austria.

## Abstract

The low-threshold L-type calcium channel Ca_v_1.3 accelerates the pacemaker rate in the heart, but its functional role for the extended dynamic range of neuronal firing is still unresolved. Here, we show that Ca_v_1.3 calcium channels act as unexpectedly simple, full-range linear amplifiers of firing rates for lateral dopamine substantia nigra (DA SN) neurons in mice. This means that they boost in vitro or in vivo firing frequencies between 2 and 50 hertz by about 30%. Furthermore, we demonstrate that clinically relevant, low nanomolar concentrations of the L-type channel inhibitor isradipine selectively reduce the in vivo firing activity of these nigrostriatal DA SN neurons at therapeutic plasma concentrations. Thus, our study identifies the pacemaker function of neuronal Ca_v_1.3 channels and provides direct evidence that repurposing dihydropyridines such as isradipine is feasible to selectively modulate the in vivo activity of highly vulnerable DA SN subpopulations in Parkinson’s disease.

## Teaser

Midbrain dopamine neuron firing amplification by the calcium channel Ca_v_1.3 suggests a treatment route for Parkinson disease.

## INTRODUCTION

The low-threshold L-type calcium channel Ca_v_1.3 has essential physiological functions including synaptic transmission in auditory hair cells and cardiac pacemaking (*1*, *2*). Neuronal Ca_v_1.3 channels have been implicated in control of synaptic plasticity (*3*, *4*) and contribution to persistent inward currents for dynamic gain control in α-motoneurons (*5*). However, their functional role for neuronal pacemaking (*6*), particularly in dopamine (DA) midbrain neurons, is still unresolved. While L-type channel function in DA neurons has been studied over the past three decades (*7*), there is still no consensus regarding the pacemaker function of Ca_v_1.3 channels and the coexpressed high-threshold L-type channel Ca_v_1.2. The reported findings of inhibiting L-type calcium channels in pacing DA neurons vary widely: from silencing of the pacemaker activity (*8*), reduction of the pacemaker rate (*9*), to no frequency effect at all (*10*) (for summary of these studies, see table S1). An interpretation of these results is further complicated by experimental differences in animals, age, recording techniques, and, in particular, the concentrations of L-type calcium channel inhibitors used. Therefore, complete silencing of pacemaking by dihydropyridines (DHPs), which inhibit L-type calcium channels by acting as negative allosteric modulators, may involve effects in addition to inhibition of L-type channels including Ca_v_1.3 (*11*). Moreover, L-type channels might have differential functions in distinct subtypes of DA neurons that reside in either the substantia nigra (SN) or ventral tegmental area (VTA) (*12*–*16*). Last, there is currently no pharmacological approach to differentiating with certainty between the high- and low-threshold L-type calcium channels Ca_v_1.2 and Ca_v_1.3 (*17*). Regarding the function of L-type channels, in contrast to cardiac pacemaking, DA neurons have an about 10-fold larger in vivo dynamic firing range (1 to 50 Hz), which is generated by boosting the autonomous pacemaking (in the range of 1 to 6 Hz) by synaptic drive into the high-frequency range. The function of L-type channels in this extended high-frequency firing range has been studied in vitro arguing for an L-type–dependent amplification of burst rate (*18*, *19*). In particular, a dynamic clamp study using modeled Ca_v_1.3 channels supported their function in burst amplification (*20*). However, neither real Ca_v_1.2 and Ca_v_1.3 channels nor distinct DA subpopulations were investigated in these studies. In addition, the role of L-type calcium channels for in vivo burst firing remains unknown [but see (*21*)]. Not only is DA burst firing known to have essential functions in reward prediction error signaling, reinforcement learning, and initiation of voluntary movement (*22*–*27*) but also recently found de novo gain-of-function mutations in *CACNA1D* (coding for Ca_v_1.3 α1 subunits) lead to neurodevelopmental disorders, including autism spectrum disorders (*28*, *29*). Last, DHPs such as isradipine have been tested for modulation of DA neurons for potential neuroprotection in Parkinson’s disease (PD) [reviewed in (*7*)]. In summary, there is great interest in identifying the functional roles of Ca_v_1.3 channels in the DA system.

To define Ca_v_1.3 channel function for both autonomic pacing and synaptically driven high-frequency firing in identified DA neurons, we make use of Ca_v_1.2DHP^−/−^ mice (*30*), where Ca_v_1.3 channels remain the only L-type channel with high DHP sensitivity in DA neurons (*31*). In addition, we combine nanomolar range DHP pharmacology with in vitro dynamic clamp approaches and with in vivo recording of identified DA SN neurons. In essence, we found a highly specific and DA subtype–selective function for Ca_v_1.3 channels acting as full-range linear amplifiers of mean firing rates in lateral nigrostriatal DA neurons. In other words, the depolarizing currents via open Ca_v_1.3 channels increase the mean discharge *x* of any of these DA neurons by a constant factor of about 1.3 for the frequency range above a Ca_v_1.3-independent baseline discharge *y* [Ca_v_1.3-mediated frequency gain = 1.3 × (*x* − *y*)].

## RESULTS

### Ca_v_1.3 channels are linear amplifiers of autonomous pacemaking in DA SN neurons

**Fig. 1.**
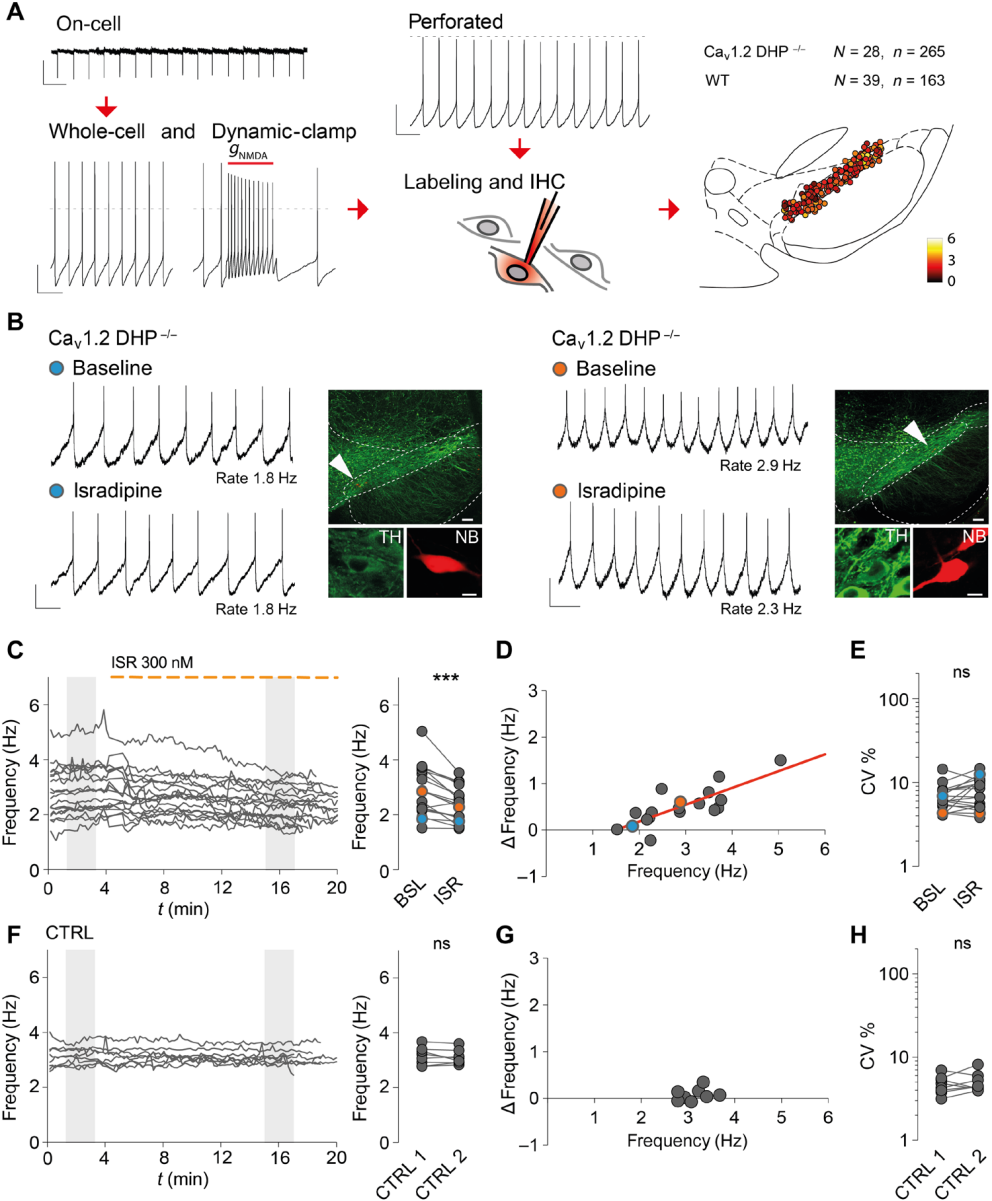
Ca_v_1.3 channels are linear amplifiers of autonomous pacemaking in DA SN neurons. (**A**) Multiple approaches for monitoring pacemaking (on-cell, whole-cell, dynamic, and perforated patch-clamp recordings). Neurons from adult Ca_v_1.2DHP^−/−^ and WT mice labeled with neurobiotin (NB) and tyrosine hydroxylase (TH) immunohistochemistry (IHC) were anatomically mapped. Dashed lines indicate membrane potential at 0 mV. Scale bars, 20 pA, 500 ms (on-cell); 20 mV, 500 ms (whole-cell); and 20 mV, 500 ms (perforated). (**B**) Left: Representative example of a perforated patch clamp–recorded DA SN neuron before and after wash in ISR (300 nM). Neuron was NB-labeled, TH-positive, and localized in the SN (white arrowheads). Scale bars, 10 mV, 500 ms (perforated) and 100 and 10 μm (histology). Right: Example DA SN neuron responding to ISR with reduction in pacemaker frequency. Note different baseline frequencies (2.9 Hz versus 1.8 Hz). Scale bars, 5 mV, 500 ms (perforated); and 100 and 10 μm (histology). (**C**) Left: Frequencies over time for DA SN neurons in Ca_v_1.2DHP^−/−^ mice. Dashed line marks the presence of ISR (*t* > 4 min). Right: Frequencies at baseline (BSL) and in isradipine (ISR). Representative examples marked with blue and orange circle, respectively. (**D**) Scatter plot showing the distribution of ISR-induced reduction of firing frequencies (Δfrequency = BSL − ISR) and corresponding baseline frequencies. A linear regression was fitted to represent the distribution (red line). (**E**) CV % at baseline and in ISR. (**F** to **H**) Data are presented as in (C) to (E) for control recordings in Ca_v_1.2DHP^−/−^ mice. ***P < 0.001. See table S2 for statistical analysis and *n* numbers. ns, not significant.

To selectively study the role of Ca_v_1.3 channels in autonomous pacemaking of DA SN neurons, we combined on-cell, whole-cell, and perforated patch-clamp recordings of labeled and identified DA SN neurons with dynamic clamp pharmacology. In addition, we compared results across two distinct genotypes, i.e., between wild-type (WT) C57BL/6 and Ca_v_1.2DHP^−/−^ mice. In the latter, DHPs selectively inhibit Ca_v_1.3 channels. Furthermore, in Ca_v_1.2DHP^−/−^ mice, we compared the role of Ca_v_1.3 in pacemaking in the presence or absence of somatodendritic D2 autoreceptor (D2 AR) signaling. In total, as depicted in Fig. 1A, the main in vitro dataset composed of *n* = 428 identified DA SN neurons from *N* = 67 mice. This was complemented by experiments on DA VTA neurons (*N* = 7, *n* = 91) and DA neurons with identified axonal projections (*N* = 6, *n* = 22).

Figure 1B shows two representative examples of perforated-patch current-clamp in vitro recordings of pacing DA SN neurons before (baseline) and after 15-min bath application of 300 nM of the L-type calcium channel inhibitor ISR (see table S1). Note the absence of firing rate change in the DA SN neuron firing at 1.8 Hz (left) by wash-in of 300 nM ISR. In contrast, the DA SN neuron firing at a faster spontaneous rate of 2.9 Hz (right) was affected by 300 nM ISR, which reduced the firing rate by about 21% (2.9 to 2.3 Hz). Figure 1C displays the firing rate before and during wash-in of 300 nM ISR of *n* = 18 identified DA SN neurons from Ca_v_1.2DHP^−/−^ mice (left). Overall, the firing rate of DA SN neurons was significantly reduced by wash-in of 300 nM ISR [mean of differences, 0.5 Hz (18%); control, mean = 2.89, SD = 0.89; ISR, mean = 2.38, SD = 0.62, *t*(17) = 5.26, *P* < 0.0001]. The degree of ISR inhibition was strongly predicted by the respective baseline firing of DA SN neurons. Those DA SN neurons firing around 2 Hz were not affected by ISR, while faster-discharging DA SN neurons within the spontaneous in vitro pacemaker range of 2 to 6 Hz were slowed down by the L-type channel inhibitor in a linear, rate-dependent fashion (Fig. 1D). In other words, the reduction of pacemaker frequencies (ΔHz) by full Ca_v_1.3 channel inhibition was positively correlated with baseline frequency (slope of the regression = 0.36; see table S2). ISR reduced the firing rate of DA SN neurons by up to 36% within the in vitro pacemaker range (fig. S1A). In contrast, the regularity of firing, expressed as the coefficient of variation (CV %), was not affected by ISR (Fig. 1E), and no correlation between CV % changes and firing rates was observed (fig. S1, C and F). Note that CV % was also used to monitor the stability of the perforated-patch configuration (fig. S1G). In a set of control recordings without wash-in of ISR, pacemaker frequency and CV % were stable over the relevant recording period (Fig. 1, F to H). In essence, our data show that Ca_v_1.3 channels act as linear amplifiers of autonomous firing rate for those DA SN neurons with spontaneous discharge above 2 Hz.

### Ca_v_1.3 channel acting as linear amplifiers predicts firing rate changes in ISR, independent of somatodendritic D2 AR or Ca_v_1.2 channels

**Fig. 2.**
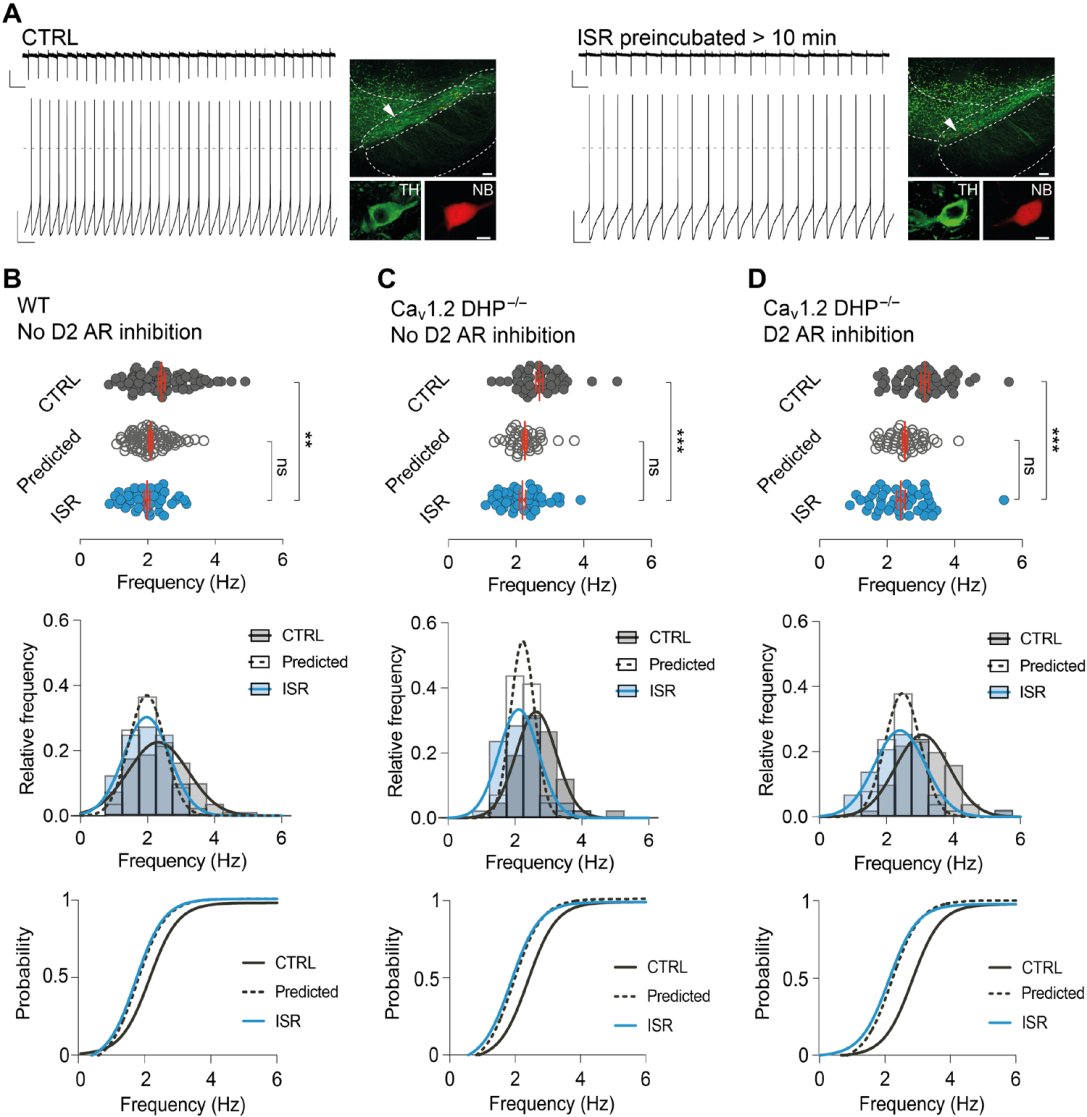
Ca_v_1.3 channel acting as linear amplifier predicts firing rate changes in ISR, independent of somatodendritic D2 AR or Ca_v_1.2 channels. (**A**) Regular pacemaking was first monitored in the on-cell (top) followed by the whole-cell recording mode (bottom). Note the stability in pacemaking frequency and regularity in both modes. Recorded DA SN neurons were labeled and histologically verified. Dashed line indicates membrane potential at 0 mV. Scale bars, 20 pA, 500 ms (on-cell); 20 mV, 500 ms (whole-cell); and 100 and 10 μm (histology). Midbrain slices were preincubated with ISR (30 or 300 nM) for over 10 min before each experiment (right). Scale bars, 20 pA, 500 ms (on-cell); 20 mV, 500 ms (whole-cell); and 100 and 10 μm (histology). (**B**) Top: Scatter plot of control, predicted, and ISR-dependent frequencies. Experiments were performed in WT mice. Middle: Frequency histograms of the respective distribution were fitted with a Gaussian function. Bottom: A left shift in the cumulative distribution marks the reduction in frequency. Note that the cumulative distribution for discharge rate in the presence of ISR is very similar to the predicted cumulative distribution. (**C**) Data for Ca_v_1.2DHP^−/−^ mice are presented as in (B). (**D**) Data for Ca_v_1.2DHP^−/−^ mice with D2 AR inhibition are presented as in (B). All data are means ± SEM. **P < 0.01, ***P < 0.001. See table S2 for statistical analysis and *n* numbers.

To test whether Ca_v_1.3 channel function as rate-dependent amplifiers of pacemaking is a general feature of DA SN neurons, we compared predicted ISR-induced rate changes with those experimentally observed after preincubation with two saturating ISR concentrations (30 and 300 nM) in three different scenarios. These two concentrations yielded very similar results, and data were pooled. Figure 2A shows representative examples where DA SN neurons were recorded initially in on-cell (see fig. S2) and subsequently in standard whole-cell configuration. Note that for these whole-cell recordings, we used the recently established in vivo patch pipette solution with adjusted internal free calcium concentrations (estimated to be about 100 nM), which preserved well the physiological discharge pattern of DA neurons (*32*). We compared experimental datasets recorded in two genotypes (WT and DHP1.2^−/−^) and in the absence or presence of D2 AR inhibition (±600 nM sulpiride) with corresponding datasets after >10-min preincubation with 30 or 300 nM ISR. As D2 AR activates G protein–gated inwardly rectifying potassium 2 (GIRK2) channels in DA SN neurons, we wanted to test whether this potassium conductance, if present, might in part shunt the pacemaker function of Ca_v_1.3.

In Fig. 2B, we plot the mean firing rate of DA SN neurons from WT mice recorded under control conditions in the absence of somatodendritic D2 AR inhibition. Here, the pacemaker frequencies ranged from about 1 to 5 Hz. We then applied the linear function describing the rate-dependent contribution of Ca_v_1.3 to pacemaker rate (as shown in Fig. 1D) to this dataset to predict the frequency distribution under selective Ca_v_1.3 inhibition. Last, we recorded firing of DA SN neurons from Ca_v_1.2DHP^−/−^ mice with or without D2 AR inhibition after preincubation with 30 or 300 nM ISR. In WT mice, in which ISR inhibits both Ca_v_1.3 and Ca_v_1.2 L-type channels, the experimental mean firing frequency distribution recorded in the presence of ISR was significantly reduced by −0.43 Hz (for statistics, see table S2), in good agreement with our quantitative prediction (mean frequency reduction = −0.33 Hz). This pharmacological effect was completely preserved in Ca_v_1.2DHP^−/−^ mice (Fig. 2C; mean frequency reduction: ISR, −0.7 Hz; predicted, −0.59 Hz), in which ISR effects mediated through Ca_v_1.2 are prevented, but Ca_v_1.3 inhibition remained unaffected. We also repeated the experiment in Ca_v_1.2DHP^−/−^ mice in the presence of D2 AR inhibition (Fig. 2D). Again, the frequency distribution after full Ca_v_1.3 inhibition was left shifted by −0.5 Hz, very similar to the quantitative prediction (−0.44 Hz). This result also indicated that D2 AR–activated GIRK2 conductance, if present, did not affect Ca_v_1.3 pacemaker function.

For better visualization of these frequency distributions, they were fitted with Gaussian functions (Fig. 2, B to D, middle) and converted to normalized cumulative distributions (Fig. 2, B to D, bottom). These findings provide direct experimental evidence that low-threshold Ca_v_1.3 channels directly control pacemaker rate and Ca_v_1.2 has no or only a minor role in pacemaker rate control in DA SN neurons. In contrast to Ca_v_1.3, which is expected to mediate continuous calcium inward current in the subthreshold range, the other L-type calcium channel expressed in DA SN neurons, Ca_v_1.2, has a higher threshold of activation and is thus likely to activate mostly during action potential firing (*9*).

To test whether the contribution of Ca_v_1.3 to pacemaker rate is also functional in neurochemically and anatomically identified DA VTA neurons, we repeated the experiments in these neurons. A total of *n* = 47 control DA VTA neurons and *n* = 44 DA VTA neurons preincubated in 30 nM ISR were recorded. In contrast to DA SN neurons, inhibition of Ca_v_1.3 had no significant effect on the firing frequency distribution of DA VTA neurons (fig. S3).

### Linear amplification of firing across the entire dynamic frequency range in lateral DA SN neurons

**Fig. 3.**
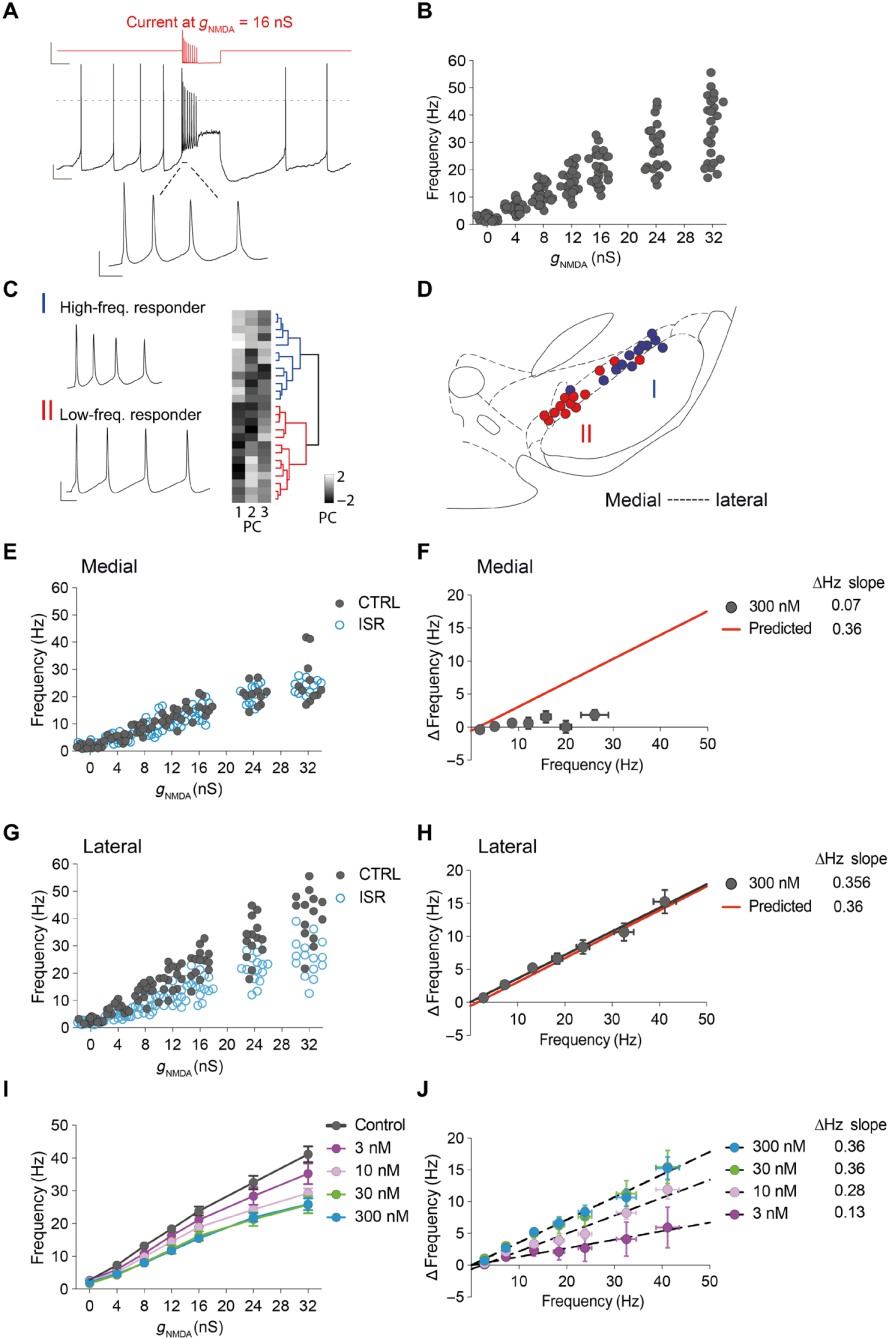
Linear amplification of firing across the entire frequency range in lateral DA SN neurons. (**A**) Somatic NMDA conductances (*g*_NMDA_) applied for expansion of in vitro firing range in DA SN neurons (WT mice) recorded in the whole-cell configuration. Red line shows current injection for *g*_NMDA_ = 16 nS. Dashed line indicates membrane potential at 0 mV. Bottom: Magnification of first three ISIs. Scale bars, 250 pA, 500 ms (top); 10 mV, 500 ms (middle); and 20 mV, 25 ms (bottom). (**B**) Scatter plot of mean frequencies averaged across the first three ISIs versus *g*_NMDA_ (*f*-*g* distribution). (**C**) Representative traces of class I (blue) and II (red) based on hierarchical clustering. Right: Rows represent individual neurons ordered according to PCs. Scale bar, 20 mV, 25 ms. (**D**) Distribution of class I and II neurons across SN. (**E**) Firing distribution of medial DA SN neurons in control and in the presence of ISR (300 nM). (**F**) Scatter plot of Δfrequency (= mean CTRL − ISR) and CTRL frequency (in hertz) for medial DA SN neurons. Linear regression line (red line) represents predicted Δfrequency. (**G**) Data are presented as in (E) for lateral DA SN neurons. (**H**) Data are presented as in (F) for lateral DA SN neurons. (**I**) *f*-*g* curves of lateral DA SN neurons in the absence (control) or presence of 3, 10, 30, and 300 nM ISR. ISR (10, 30, and 300 nM) caused significant inhibition of frequency versus control at all *g*_NMDA_ intensities [repeated-measures two-way analysis of variance (ANOVA), Tukey’s multiple comparisons test]. (**J**) Gradual decrease in ΔHz slope with clinically relevant low nanomolar concentrations of ISR. Slopes were significantly different from zero and from each other (except 30 from 300 nM ISR). All data are means ± SEM. See table S2 for statistical analysis and *n* numbers.

Next, we asked whether Ca_v_1.3 also controls higher discharge frequencies above the intrinsic pacemaker rate. The extended frequencies are observed in vivo and range from about 1 to 50 Hz in identified DA SN neurons (*13*). We applied the dynamic clamp technique (*33*) to drive WT DA SN neurons in vitro into this frequency range by injecting increasing amounts of *N*-methyl-d-aspartate receptor (NMDAR) conductance (*g*_NMDA_ = 0 to 32 nS; Fig. 3A). Mean *g*_NMDA_-evoked firing frequencies of *n* = 25 (*N* = 8) individual DA neurons located across the entire mediolateral extent of the SN were plotted against increasing NMDAR conductances (Fig. 3B). Note the high variability of evoked firing frequencies [i.e., the mean frequencies averaged across the first three interspike intervals (ISIs) as a function of *g*_NMDA_; see figure legend], particularly at large *g*_NMDA_ ranging from 10 to 50 Hz. To gain further insight into this variability, we performed a principal components analysis on *g*_NMDA_-induced firing properties (see Methods). The first three principal components (PC1 to PC3; Fig. 3C) accounted for almost 85% of the variance and were thus used for unsupervised hierarchical clustering (fig. S4). Two separate populations emerged from this cluster analysis, with cluster I representing high frequency–responding and cluster II low frequency–responding DA SN neurons, respectively (Fig. 3C). Mapping these DA SN neurons across the mediolateral axis of the SN revealed a clear anatomical segregation, with high-frequency responders clustering more in the lateral and low-frequency responders clustering more in the medial aspect of the SN (Fig. 3D). As our recent in vivo study of retrogradely identified DA SN neurons revealed similar functional differences between dorsomedial striatum (DMS)–projecting DA neurons in the medial and dorsolateral striatum (DLS)–projecting DA neurons in the lateral SN neurons (*13*), we repeated the dynamic clamp experiments on retrogradely identified DLS-lateral SN and DMS-medial SN DA neurons (fig. S5). These data revealed that DLS-lateral SN DA neurons responded to *g*_NMDA_ conductances with significantly higher evoked mean firing rates as compared to DMS-projecting DA neurons in the medial SN (fig. S5, B and C), very similar to the difference seen between lateral and medial nontraced neurons (fig. S5D). Together, these results suggested a more prominent role of L-type channels in the high-frequency range for DLS-projecting DA neurons in the lateral SN. When we repeated the dynamic clamp experiments for lateral and medial DA SN neurons, we found that the evoked firing frequencies of medial DA SN neurons in the presence of 300 nM ISR were not different from controls (Fig. 3E) and therefore violated the predicted contribution of Ca_v_1.3 channels (Fig. 3F, compare Fig. 1D). In contrast, the dynamic clamp–evoked high-frequency firing of DA SN neurons in the lateral SN in the presence of 300 nM ISR was significantly lower compared to controls (Fig. 3G). A closer inspection revealed that lateral DA SN neurons in ISR displayed similar firing rates to medial DA SN neurons under control conditions (fig. S6C). However, other firing properties distinct between medial and lateral DA SN neurons, such as minAHP (minimum after hyperpolarisation potential), spike threshold, and spike width remained significantly different (fig. S6, D to F). The degree of frequency reduction by ISR in lateral DA SN neurons was very similar to that quantitatively predicted by the linear amplification function of Ca_v_1.3 (Fig. 3H, compare Fig. 1D). This indicated that Ca_v_1.3 channels linearly amplify the firing rate by about 30% across the entire dynamic firing range in lateral DA SN neurons. In contrast, the full-range firing of medial DA SN neurons was not affected by ISR. We further investigated this different drug responsiveness between medial and lateral DA SN neurons, which might be caused by differential expression of Ca_v_1.3 channels and by differential coupling to calcium-activated potassium channels. Inhibition of apamin-sensitive Ca^2+^-activated K^+^ (SK) channels exerted a differential effect on the discharge pattern and rate of putative DA SN neurons in vitro (*34*), which early on already suggested the existence of functionally distinct DA subpopulations (*35*). Therefore, we used the dynamic clamp protocol in the presence of 300 nM of apamin to test whether SK channels are differentially involved in controlling the full firing range (fig. S7). In contrast to control conditions, the *f*-*g* distributions of lateral and medial DA SN neurons were not different in apamin (fig. S7, B and C). Furthermore, this effect was mainly driven by a significant apamin-induced increase in excitability in the medial DA SN subpopulation (see fig. S7C). In contrast, inhibition of SK channels in lateral DA SN neurons did not significantly enhance firing excitability. These findings demonstrated that negative feedback mechanism via SK channels is selectively operative in medial DA SN neurons. We then tested whether preventing SK channel feedback via enhanced calcium buffering [pipette solution with 1 mM 1,2-bis(2-aminophenoxy)ethane-*N*,*N*,*N*′,*N*′-tetraacetic acid (BAPTA)] would unmask potential Ca_v_1.3-mediated amplification of firing rates in medial DA SN neurons (fig. S8). As expected, firing frequencies of medial DA SN neurons in 1 mM BAPTA were very similar compared to those in apamin (fig. S8, A to C, compare with fig. S7C). However, addition of ISR had no additional effects on discharge rates in medial DA SN neurons (fig. S8D). These results confirm that Ca_v_1.3 channels have no contribution to full-range firing in medial DA SN neurons and suggest that, in additional contrast to lateral DA SN neurons, other voltage-gated calcium channels [e.g., N-type calcium channels (*36*)] provide powerful SK-mediated negative feedback on full-range firing excitability. Moreover, the experiments in 1 mM BAPTA revealed that the degree of Ca_v_1.3-mediated amplification in lateral DA SN neurons was partially responsive to changes in internal calcium buffering. As shown in fig. S8 (E to G), the ISR-sensitive linear amplification slope was about 25% smaller in 1 mM BAPTA compared to the control pipette solution containing 0.1 mM EGTA calcium buffering (1 mM BAPTA + ISR, 0.28; predicted from 0.1 mM EGTA, 0.36; fig. S8H). This difference might have mechanistic implications in the sense that about 25% of the Ca_v_1.3-mediated amplification were mediated by more indirect, permissive effects (e.g., direct or enzyme-mediated calcium regulation of other channels, transporters, or pumps) and thereby easily perturbed by changes in calcium buffering. However, the dominant component of Ca_v_1.3-mediated amplification—not affected by 1 mM BAPTA—is more likely to result from a direct electrogenic effect of Ca^2+^—influx via open Ca_v_1.3 channels.

To further differentiate these two mechanistic contributions of Ca_v_1.3 to the firing gain, we again used the dynamic clamp as a way to drive DA neurons electrogenically. To assess the level of endogenously active Ca_v_1.3 conductance in individual lateral DA SN neurons, we initially titrated the amount of Ca_v_1.3 anticonductance (anti-*g*_Ca_v_1.3_; see Methods) to reduce spontaneous firing by about 0.35, which corresponded to the experimentally determined amplitude of Ca_v_1.3 firing gain (fig. S9, A and B). Subsequently, we compared the *g*_NMDA_-mediated firing distribution of lateral DA SN neurons in the presence or absence of the approximated Ca_v_1.3 anticonductance (*n* = 6; fig. S9, C and D). We found that the activation of single cell–titrated Ca_v_1.3 anticonductance reduced the gain by about 0.28 (fig. S9, E to G, compare with fig. S8H). This is very similar to the gain of biological Ca_v_1.3 channels in 1 mM BAPTA but less than the full Ca_v_1.3 gain described above. This result is in accordance with the limitations of the dynamic clamp method, which at best can only approximate the direct electrogenic effect of a conductance. In summary, we presented evidence that most of the Ca_v_1.3-mediated firing gain is driven electrogenically. Furthermore, the Ca_v_1.3 gain is effective in lateral DA SN neurons given their low degree of SK-mediated negative feedback.

These in vitro experiments have important and testable implications for the role of Ca_v_1.3 channels on the in vivo firing of DA SN subpopulations, where we would expect a more pronounced, if not selective, effect on lateral DA SN neurons. However, before exploring in vivo physiology, we quantified the concentration-dependent effects of ISR on Ca_v_1.3 inhibition in a low nanomolar range, including therapeutic peak plasma concentrations reported in humans [8 to 16 nM for a dose of 5 mg of ISR, (*37*, *38*)]. As shown in Fig. 3 (I and J), ISR concentration dependently reduced *g*_NMDA_-evoked firing frequencies of lateral DA SN neurons with a saturating effect at 30 nM and a strong and significant inhibition at 10 nM, a concentration well within the clinical range. Moreover, the inhibition by 3 to 10 nM ISR could also be described by linear functions (significantly different from zero; see table S2), which had a smaller slope (3 nM, 0.13; 10 nM, 0.28) compared to those obtained under saturating ISR concentrations (30 and 300 nM, 0.36). On the basis of these in vitro results, we were able to make distinct quantitative predictions for the ISR effect on the firing frequency of medial and lateral DA SN neurons in the intact brain.

### ISR (10 nM) reduces in vivo firing frequencies in lateral DA SN neurons

To study the effect of clinically relevant concentrations of ISR in vivo, we recorded the spontaneous activity of identified DA SN neurons in isoflurane-anesthetized 2- to 3-month-old WT mice. ISR was injected intraperitoneally at a dose of 3 mg/kg, which was previously shown to result in similar plasma concentrations in the range as reported for patients taking therapeutic doses (*37*, *38*). Recorded DA neurons were juxtacellularly labeled for post hoc immunohistochemical identification and anatomical localization within the SN. In addition, DA SN neurons, for which labeling was insufficient for detection, were functionally identified by their broad spike widths and localized according to the track of the recording electrode targeting either the medial or lateral SN (see Methods and fig. S10).

**Fig. 4.**
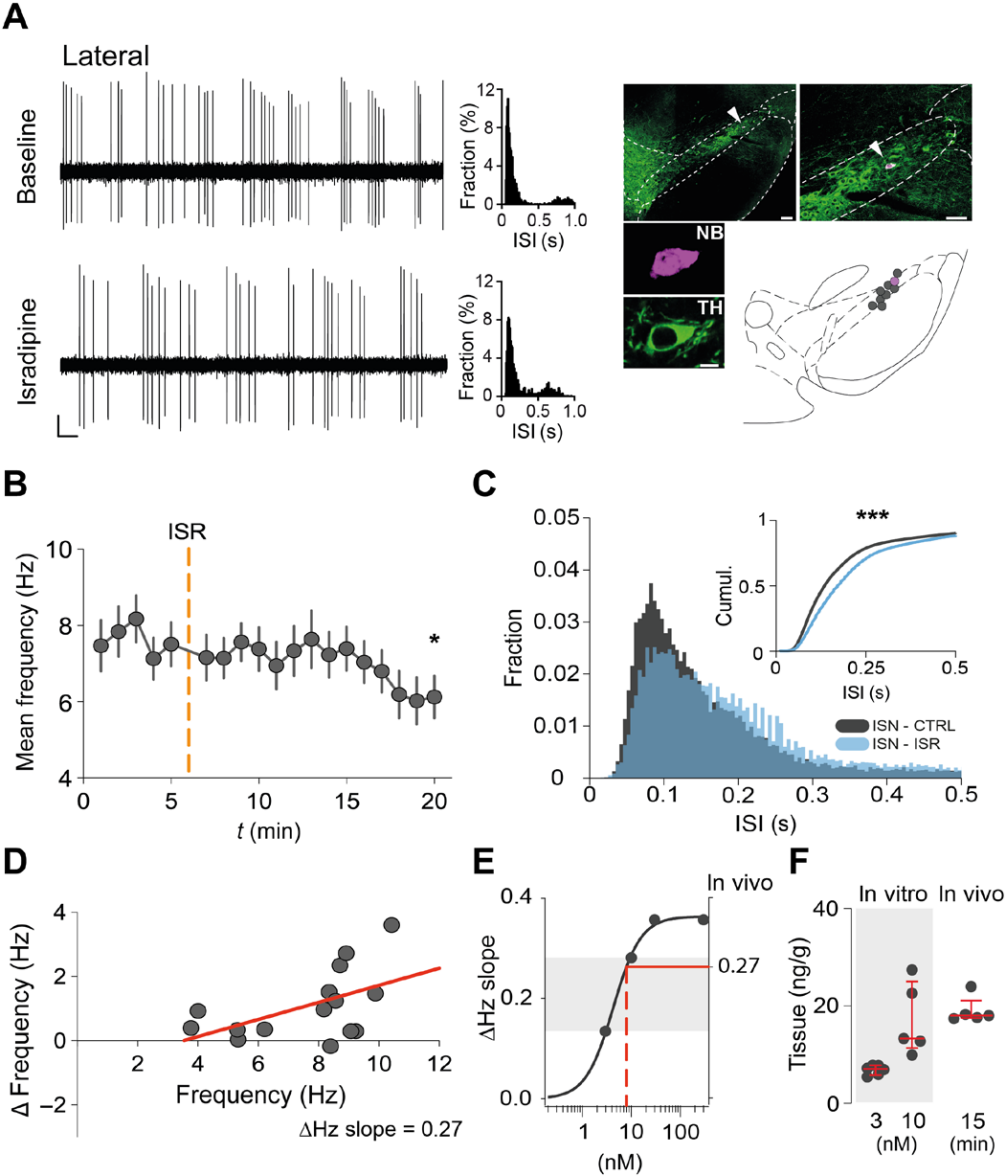
ISR (10 nM) reduces in vivo firing frequencies in lateral DA SN neurons. (**A**) Left: Representative trace of a lateral DA SN neuron before and after intraperitoneally injecting ISR (3 mg/kg). Note the suppression of firing activity after administration of systemic ISR. Middle: Normalized histograms of ISIs at baseline and in ISR (5 min before and last 5 min after ISR injection, respectively). Right: Recorded neuron was juxtacellularly labeled with NB for histological verification and localization in the SN. Scale bars, 0.2 mV, 500 ms (extracellular recording); and 100, 50, and 10 μm (histology). (**B**) Progression of mean frequency during 20 min of recorded firing activity. Orange dashed line (*t* = 6 min) marks injection of ISR. *n* = 15, *N* = 15 (**C**) Overlay of ISI histograms at baseline (5 min before injection) and in ISR (10 to 15 min after injection). Inset: Cumulative distributions of the ISI histograms. For better visualization, only ISIs of <0.5 s are depicted. (**D**) Distribution of Δfrequency and baseline frequency (in hertz) for each DA SN neuron. A linear regression line with a slope of 0.27 was fitted (red line). (**E**) A concentration-response curve with ΔHz slopes plotted against the indicated ISR concentrations (see Fig. 3). A ΔHz slope of 0.27 (horizontal, red line) predicts an effective concentration of ISR at around 8 nM (vertical, red dashed line). (**F**) Tissue concentrations in acutely prepared midbrain slices after preincubation in 3 or 10 nM ISR, respectively, were compared with tissue concentrations of midbrains 15 min after systemic application of ISR (3 mg/kg). All data are means ± SEM. *P < 0.05, ***P < 0.001. See table S2 for statistical analysis and *n* numbers.

Figure 4A shows representative extracellular in vivo recording from an identified DA neuron in the lateral SN at baseline (top left) and 10 and 15 min after ISR injection (bottom left). Note the reduction of firing frequency in ISR as compared to baseline, which is also apparent in the ISI distribution (middle, top, and bottom). The in vivo firing activities of identified lateral DA SN neurons (combined immunohistochemical and functional identification: *n* = 15, *N* = 15) were characterized by a mean firing frequency of around 7 Hz. Before systemic administration of ISR (3 mg/kg), baseline activity was recorded for about 5 min. After ISR injection, we observed a gradual reduction of the mean frequency of lateral DA SN neurons over the first 15 min when the recording was terminated for juxtacellular labeling (Fig. 4B). Compared to baseline, the ISI histogram of the lateral DA SN population recorded 10 to 15 min after systemic ISR injection showed a significant 32-ms shift to longer ISI durations at a cumulative probability of 0.5 (Fig. 4C).

As shown in Fig. 4D and similar to our in vitro data, we plotted the relative ISR effect on mean firing rates for each of the identified *n* = 15 lateral DA SN neuron against their respective baseline value. This distribution was well described with a linear regression and a ΔHz slope of 0.27. As evident from the concentration-response curve shown in Fig. 4E, a ΔHz slope of 0.27 (horizontal, red solid line) is very similar to the one observed for our in vitro dynamic clamp data in the presence of 10 nM ISR (Fig. 4E; see also Fig. 3J), indicating that inhibition of Ca_v_1.3 channels by pharmacologically relevant low nanomolar ISR concentrations is sufficient to explain the in vivo reduction of firing frequency in lateral DA SN neurons. For further experimental support of this notion, we quantified the ISR tissue concentrations found in vivo in the relevant time window for recording using liquid chromatography–tandem mass spectrometry (LC-MS/MS; see Methods) and compared it to the in vitro midbrain slice concentrations achieved by preincubation of midbrain samples with 3 and 10 nM ISR. This revealed a median tissue concentration of 7 ng/g [interquartile range (IQR), 5.7 to 7.8] and 13.3 ng/g (IQR, 11.3 to 25), respectively. The median midbrain tissue concentration within the in vivo recording time [15 min after intraperitoneal injection of ISR (3 mg/kg)] was 18 ng/g (IQR, 17.5 to 21.12) and therefore in a similar range as determined for the in vitro slice tissue concentration after 10 nM preincubation of ISR (Fig. 4F). Thus, the measured concentrations validate the comparison of in vitro and in vivo effects of ISR on firing frequencies in lateral DA SN neurons.

### ISR (10 nM) does not affect in vivo firing properties of medial DA SN neurons

**Fig. 5.**
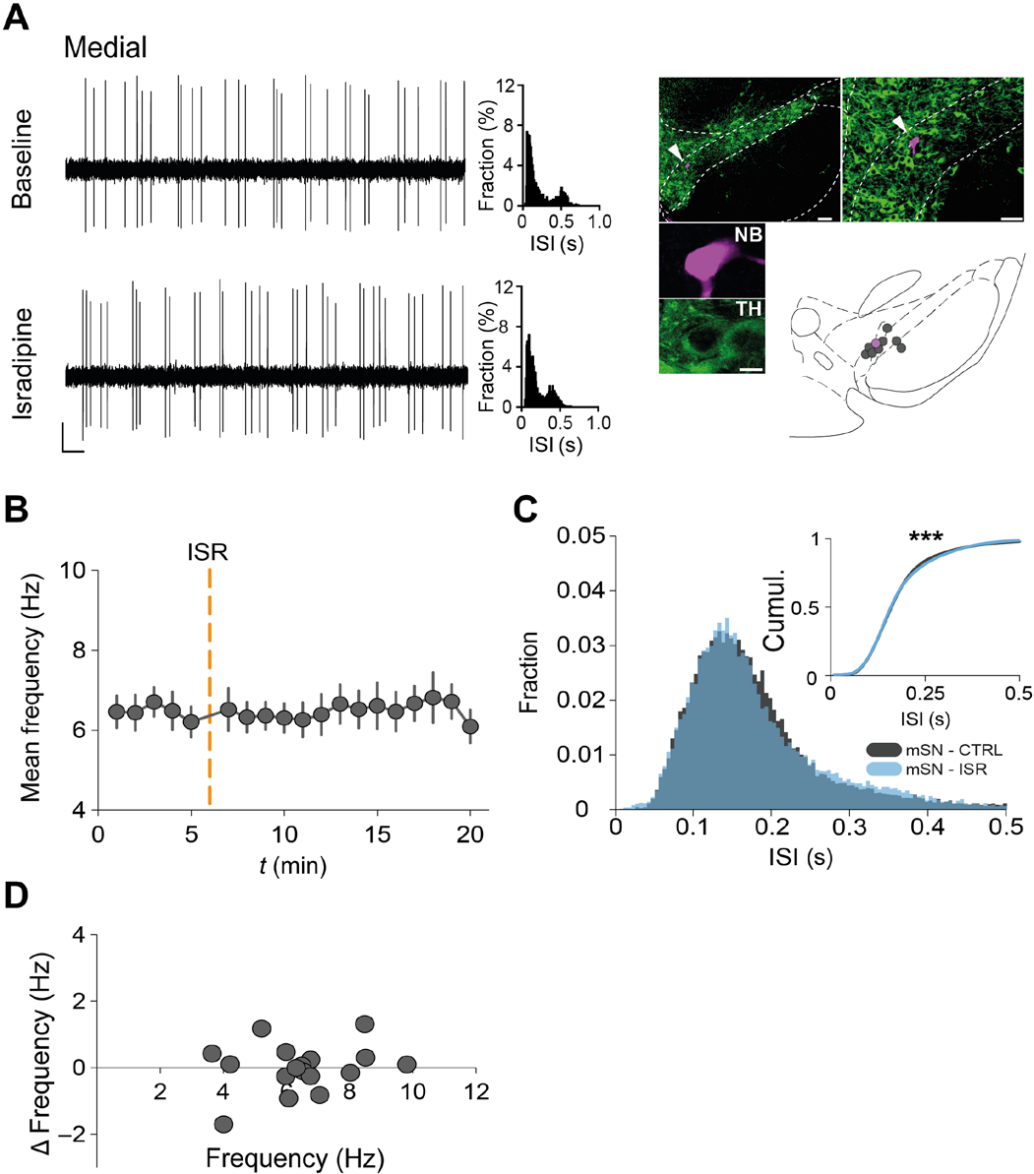
ISR (10 nM) does not affect in vivo firing properties of medial DA SN neurons. (**A**) Left: Representative traces of a medial DA SN neuron before and after intraperitoneally injecting ISR (3 mg/kg). Middle: Normalized ISI histograms. Right: Target neuron was juxtacellulary labeled, histologically verified, and localized in the SN. Scale bar, 0.2 mV, 500 ms (extracellular recording); and 100, 50, and 10 μm (histology). (**B**) Progression of mean frequency during 20 min of recorded firing activity. Orange dashed line (*t* = 6 min) mark injection of ISR. (**C**) Overlay of ISI histograms at baseline and in ISR. Inset: Cumulative distributions of the ISI histograms. For better visualization, only ISIs of <0.5 s are depicted. Note that while statistical significance is met using a two-sample Kolmogorov-Smirnov test, the absolute shift at cumulative probability = 0.5 is <1 ms (see table S2 for test statistics). (**D**) Distribution of Δfrequency and baseline frequency (in hertz) for each DA SN neuron. All data are means ± SEM. ***P < 0.001. See table S2 for statistical analysis and *n* numbers.

Our in vitro dynamic clamp experiments demonstrated that intrinsic Ca_v_1.3 channels are not involved in full-range firing frequency control of medial DA SN neurons. We also carried out the in vivo ISR experiment described above for identified medial DA SN neurons. Consistent with the in vitro results, the mean firing properties of medial DA SN neurons were not affected by low nanomolar concentrations of ISR (Fig. 5). In summary, the in vivo experiments revealed that lateral DA SN neurons in mice can be selectively targeted by clinically relevant low nanomolar ISR concentrations.

## DISCUSSION

In this study, we show that low threshold Ca_v_1.3 L-type calcium channels (*7*) serves as simple linear amplifiers with little negative feedback over the entire dynamic firing range in nigrostriatal DA neurons located in the lateral SN. This function is reminiscent to a full-range linear amplifier (low gain, type A) in electronics and might serve as a tool for multiplicative gain control—a fundamental feature of neural computation (*39*). Recent studies in nonpacemaking neurons identified short-term synaptic depression and inhibition by GABA (gamma aminobutyric acid) as other important elements for gain control (*40*, *41*). In α-motoneurons, Ca_v_1.3 has been suggested to contribute to nonlinear gain control (*42*, *43*). In contrast, mechanisms of gain control for pacemaker neurons have not yet been experimentally defined but probed in a modeling study (*44*).

We also demonstrate that clinically relevant, low nanomolar concentrations of the L-type channel inhibitor ISR (i.e., negative allosteric modulator) selectively reduced the in vivo firing activity of these lateral DA SN neurons. By comparing the effects of nanomolar concentration of ISR on DA SN neurons from WT and Ca_v_1.2DHP^−/−^ mice, we demonstrate that autonomous pacemaker rate control in DA SN neurons is selectively mediated by Ca_v_1.3 calcium channels with no detectable contribution of coexpressed high-threshold Ca_v_1.2. We used the dynamic clamp to explore the effects of Ca_v_1.3 channels across the entire dynamic firing range that DA SN neuron display in the intact brain. The high-frequency end of this dynamic range is realized in vivo by synaptically driven transient burst firing up to about 50 Hz. Here, we found an unexpected selectivity, as Ca_v_1.3 channels only amplified the high-frequency firing range in lateral DA SN neurons but had no effect on the electrical activity of medial DA SN neurons. Even when the substantial SK channel–mediated negative feedback was removed in medial DA SN neurons, Ca_v_1.3 channels did not contribute to the firing gain. Mechanistically, we presented evidence that direct calcium influx via open Ca_v_1.3 channels were most relevant for the firing gain in lateral DA SN neurons, while more indirect, permissive calcium effects downstream of channel function (e.g., electrogenic calcium-sodium exchangers or calcium-dependent enzymes) contributed to about 25% of the total gain.

Our in vivo experiments on identified DA SN neurons confirmed this selectivity at clinically relevant low nanomolar concentrations of ISR. In general, our study provides evidence that in vitro dynamic clamp experiments with clinically relevant concentrations of ISR can, at least under favorable conditions, be successful in accurately predicting cell type–selective in vivo drug effects.

However, our study has also some limitations. In particular, our dynamic clamp approach does only approximate realistic synaptic activity. Real glutamatergic inputs are more complex as they are defined by, e.g., specific gating kinetics, spatiotemporal distribution of synapses across the dendritic tree and, maybe most relevant here, considerable calcium influx via open NMDARs. It has been shown that calcium influx via NMDARs activates SK channels (*45*), which are also present in DA neurons (*46*). Here, we have limited our experiments to SK channel feedback on evoked firing activity but have not explored its potential roles in biological synapses. We have also not studied the regulation of native Ca_v_1.3 channels by calcium (*47*–*49*). Another limitation might be the lack of chronic ISR experiments (*9*, *50*). However, Guzman and colleagues (*50*) showed that 7- to 10-day ISR treatment did not alter the mRNA expression levels of several voltage-gated calcium channels, including Ca_v_1.3, and did not affect in vitro firing rates of DA SN neurons. In contrast, the effect of chronic in vivo ISR in the low nanomolar range on in vivo firing activity of defined DA neurons in the lateral SN remains unknown. Given the context of PD, it would also be interesting to ask whether Ca_v_1.3 function changes with aging in defined DA SN neuronal subpopulations. In this context, Branch and colleagues (*51*) demonstrated an about threefold reduction of global L-type calcium channel currents associated with a 25% reduction of pacemaker rate, while a specific role of Ca_v_1.3 was not explored. Last, we have not yet investigated potential behavioral effects of ISR-induced firing rate reductions in DA neurons in the lateral SN. Reported by da Silva and colleagues (*23*), about 50% DA SN neurons in freely moving mice generate transient increases in firing rates time locked to movement initiation. Furthermore, optogenetic activation of DA SN neurons accelerated these movements arguing for a causal link between transient DA SN firing rates and movement kinematics (*23*). Similar observations but with a predominance of movement-associated transient reductions of DA SN firing have been made in head-fixed mice (*52*). These electrophysiological findings are also supported by axonal calcium imaging of nigrostriatal DA neurons, where voluntary initiation of locomotion was preceded by increased axonal calcium signals [as a proxy for enhanced electrical activity (*24*)]. On the basis of these studies, we predict that nanomolar concentrations of ISR might also dampen movement-related firing increases and thereby potentially slow movement initiation. This could also be tested in healthy human individuals, patients with hypertension chronically treated with ISR (*53*), and in patients with PD (*37*), who already show reduced velocity during self-initiated stepping (*54*).

If we extrapolate from our acute in vivo experiments, then ISR therapy has the potential to chronically reduce the entire spectrum of firing activity of most vulnerable DA SN neurons. In mice, this DA subpopulation is found in the lateral aspect of the SN, which corresponds to the ventrolateral DA SN neurons in humans (*55*). If true, then a chronic reduction of in vivo firing rates might contribute to the significantly reduced risk (−22%) to develop PD as observed in hypertensive patients treated with DHP Ca^2+^ channel inhibitors (*53*). This notion would be in line with a substantial body of work (“stressful pacemaker hypothesis”) demonstrating that L-type channel inhibition in DA SN neurons reduces oxidative stress, mitochondrial and lysosomal dysfunctions, and α-synuclein toxicity (*8*, *14*, *56*, *57*), which are all established drivers of PD pathogenesis (*58*).

There is convincing evidence from recent pathological (*59*) and imaging studies (*60*) that the most vulnerable DA SN subpopulation is already severely damaged when PD is first clinically diagnosed. This might imply that at this time point, only a limited number of ISR-sensitive DA SN neurons are still viable. Consequently, the ISR effect on the population of PD-surviving DA neurons might be much smaller. In addition, ISR concentrations might not have been maintained at high enough brain concentration for a significant part of the day (*61*). The dose dependency of dihydropiridines in reducing the risk for PD has recently been documented in a population-based retrospective cohort study by Tseng *et al.* [(*62*)]. These factors may have contributed to the so far limited effects (*63*) of a recent clinical trial where a disease-slowing effect of ISR in newly diagnosed patients with PD was studied (*37*). In essence, our study established a defined role of Ca_v_1.3 channels in regulating neuronal activity for DA neurons in the lateral SN and demonstrated the selective in vivo targeting of these highly vulnerable neurons with clinically established concentrations of L-type calcium channel inhibitors.

## METHODS

### Animals

Male C57Bl6/N mice (Charles River Laboratories) were used for the study. Ca_v_1.2DHP^−/−^ mice carry a mutation (T1066Y, Exon 24) in the DHP-binding site of the α1 subunit rendering Ca_v_1.2 insensitive to DHPs (*30*). Mice were between 8 and 14 weeks old, group-housed, and maintained on a 12-hour light/dark cycle.

### LC-MS/MS analysis of ISR

For the analysis of ISR, samples were prepared as follows: Tissue samples were homogenized in 300 μl of water with four zirconium oxide grinding balls using a swing mill (25 Hz, 3 min; Retsch, Haan, Germany). A total of 20 μl of the plasma samples were mixed with 80 μl of phosphate-buffered saline (PBS). All samples were then spiked with 20 μl of methanol, 20 μl of the internal standard solution (ISR-d3 in methanol, TRC), and 600 μl of ethyl acetate. Afterward, samples were vortexed and centrifuged at 20,000*g* for ca. 5 min. The organic phase was removed, and the extraction was repeated with 600 μl of ethyl acetate. The organic fractions were combined and evaporated at a temperature of 45°C under a gentle stream of nitrogen. The residues were reconstituted with 50 μl of acetonitrile/water/methanol (3:6:1, v/v/v) and transferred to glass vials. For calibration standards and quality control samples, 20 μl of plasma were mixed with 80 μl of PBS, spiked with standard working solutions, and processed like the samples, starting from spiking with the internal standard.

The LC-MS/MS analysis was carried out using an Agilent 1290 Infinity LC system (Agilent, Waldbronn, Germany) coupled to a hybrid triple quadrupole linear ion trap mass spectrometer QTRAP 6500+ (Sciex, Darmstadt, Germany) equipped with a Turbo V source operating in positive electrospray ionization mode. The chromatographic separation was carried out using a Mercury Hydro-RP column (20 mm by 2.0 mm, 2.5-μm particle size, and 80-Å pore size; Phenomenex, Aschaffenburg, Germany) and maintained at 25°C. A gradient program was used at a flow rate of 400 μl/min from 0 to 0.2 min and from 3.6 to 5.5 min. A flow rate of 500 μl/min was used from 0.3 to 3.5 min. Mobile phase A was 0.0025% formic acid, and mobile phase B was acetonitrile/isopropyl alcohol/acetone 7:2:1 (v/v/v) + 0.05% formic acid + 1 mM ammonium formate. The gradient program started with 80% mobile phase A for 0.3 min, and then mobile phase A was decreased to 0% within 1.7 min and held for 1.5 min. Within 0.1 min, the initial conditions were restored, and the column was reequilibrated for 1.9 min. Total run time was 5.5 min, and the injection volume was 10 μl. Mass spectrometric parameters were set as follows: ionspray voltage, 4500 V; declustering potential, 30 V; ion source temperature, 400°C; curtain gas, 45 psi; nebulizer gas, 60 psi; turbo heater gas, 60 psi; and collision gas, 9 psi. Both quadrupoles were running at unit resolution.

For analysis and quantification, Analyst 1.6.3 software and MultiQuant 3.0.2 software (both Sciex, Darmstadt, Germany) were used. The precursor-to-product ion transition mass/charge ratio of 372.1 to 340.1 was used for quantification of ISR (collision energy, 14 V). The peak area of ISR was corrected by the peak area of the internal standard ISR-d3. Calibration curves were constructed using linear regression with 1/× weighting. The coefficient of correlation was at least 0.99. Variations in accuracy were less than 15% over the whole range of calibration, except for the lowest limit of quantification, where a variation in accuracy of 20% was accepted.

### In vivo electrophysiology

In vivo extracellular single-unit activities of DA SN neurons were recorded in mice, which were connected to an intraperitoneal line. ISR was dissolved in vehicle [corn oil/dimethyl sulfoxide (DMSO), 1.6%] at a concentration of 3 mg/kg and delivered through the line. Mice were anesthetized (isoflurane: induction, 5.0%; maintenance, 1 to 2% (>70% of recordings at 1.2%) in 0.35 liter/min of O_2_) and placed into a stereotactic frame. Craniotomies were performed to target lateral SN (bregma, −3.08 mm; lateral, 1.4 mm; ventral, 3.5 to 4.5 mm) and medial SN (bregma, −3.08 mm; lateral, 0.9 mm; ventral, 4.2 to 5.0 mm). Borosilicate glass electrodes (10 to 25 megohm; G120F-4, Harvard Bioscience, Holliston, MA, USA) were made using a horizontal puller (DMZ-Universal Puller, Zeitz, Germany) and filled with 0.5 M NaCl, 10 mM HEPES (pH 7.4), and 1.5% neurobiotin (NB; Vector Laboratories, Burlingame, CA, USA). A micromanipulator (SM-6, Luigs and Neumann, Ratingen, Germany) was used to lower the electrodes to the recording site. The single-unit activity of each neuron was first recorded for 5 min (baseline) at a sampling rate of 12.5 kHz before injection of ISR for 1 min, followed by continuous recording for 14 min. Occasionally, electrophysiological noise was caused during the injection; thus, data at *t* = 6 min were excluded from all analysis. Signals were amplified 1000× (ELC-03M, NPI Electronics, Tamm, Germany), notch- and band pass–filtered 0.3 to 5000 Hz (single-pole, 6 dB/octave; DPA-2FS, NPI Electronics), and recorded on a computer with an EPC-10 A/D converter (HEKA Elektronik, Lambrecht, Germany). Simultaneously, the signals were displayed on an analog oscilloscope and an audio monitor (HAMEG Instruments CombiScope HM1508, AUDIS-03/12 M, NPI Electronics). DA SN neurons were initially identified by their broad biphasic action potential (spike width > 1.5-ms duration) and slow frequency (1 to 8 Hz). Spike width was determined as the interval between the start of initial upward component and the minimum of following downward component (fig. S5).

### In vivo juxtacellular labeling

DA SN neurons were labeled after recording with NB using the juxtacellular in vivo labeling techniques (*64*). Microiontophoretic currents were applied (1- to 10-nA positive current, 200-ms on/off pulse, ELC-03 M, NPI Electronics) via the recording electrode in parallel to monitoring single-unit activity. Labeling was considered successful when the firing pattern of the neuron was modulated during current injection (i.e., increased activity during on-pulse and absence of activity during off-pulse), and the process was stable for at least 20 s, followed by the recovery of spontaneous activity.

### Retrograde tracing

Mice were anesthetized using isoflurane (induction, 3.5%; maintenance, 0.8 to 1.4% in O_2_, 0.35 liter/min; AbbVie) and placed in a stereotaxic frame (Kopf). Lidocaine gel was used as a local analgesic on the incision site. Body temperature (33° to 36°C) and respiration (1 to 2 Hz) were monitored continuously. Craniotomies were performed using a stereotaxic drill (0.75 mm in diameter) to target the DLS (bregma, 0.74 mm; lateral, 2.2 mm; ventral, 2.6 mm) and DMS (bregma, 0.74 mm; lateral, .2 mm; ventral, 2.6 mm). Coordinates were corrected as reported before (*16*). Red beads (200 nl; Lumaflor) diluted (1:30) in artificial cerebrospinal fluid (ACSF; Harvard Apparatus) were infused into the target area using a micropump (10-μl nanofil syringe, 35-gauge steel needle, flow rate of 100 nl/min; UMP3-1, World Precision Instruments). Patch-clamp experiments were performed 2 to 4 days after injection.

### Slice preparation

Animals were anesthetized by intraperitoneal injection of ketamine (250 mg/kg; Ketaset, Zoetis) and medetomidine hydrochloride (2.5 mg/kg; Domitor, OrionPharma) before intracardial perfusion using ice-cold ACSF consisting of the following: 50 mM sucrose, 125 mM NaCl, 2.5 mM KCl, 25 mM NaHCO_3_, 1.25 mM NaH_2_PO_4_, 2.5 mM glucose, 6 mM MgCl_2_, 0.1 mM CaCl_2_, and 2.96 mM kynurenic acid (Sigma-Aldrich), oxygenated with 95% O_2_ and 5% CO_2_. Rostral coronal midbrain slices (bregma, −2.92 to −3.16 mm) were sectioned into 250-μm slices using a vibrating blade microtome (VT1200s, Leica). Before the experiment, slices were kept at 37°C for 1 hour in oxygenated extracellular solution containing the following: 22.5 mM sucrose, 125 mM NaCl, 3.5 mM KCl, 25 mM NaHCO_3_, 1.25 mM NaH_2_PO_4_, 2.5 mM glucose, 1.2 mM MgCl_2_, and 1.2 mM CaCl_2_.

### In vitro patch-clamp recordings

#### Whole-cell current clamp

Slices were placed in a heated recording chamber (37°C) that was perfused with the oxygenated extracellular solution with a flow rate of 2 to 4 ml/min. 6-Cyano-7-nitroquinoxaline-2,3-dione (20 μM), gabazine (4 μM; SR95531), and DL-AP5 (10 μM) were added to inhibit excitatory and inhibitory synaptic transmission. Sulpiride (600 nM) was used to inhibit D2 AR (mentioned separately, when used). Neurons were visualized using infrared differential interference contrast videomicroscopy with a digital camera (VX55, Till Photonics) connected to an upright microscope (Axioskop 2, FSplus, Zeiss). Retrogradely labeled neurons were visualized by epifluorescence (X-cite 120PC Q, Excelitas Technologies) for detection of retrobeads. Patch pipettes were pulled from borosilicate glass (GC150TF-10, Harvard Apparatus, Holliston, MA, USA) using a temperature-controlled, horizontal pipette puller (DMZ-Universal Puller, Zeitz). Patch pipettes (4 to 6 megohm) were filled with a solution containing the following: 135 mM K-gluconate, 5 mM KCl, 10 mM HEPES, 0.1 mM EGTA, 5 mM MgCl_2_, 0.075 mM CaCl_2_, 5 mM Na adenosine 5′-triphosphate, 1 mM Li guanosine 5′-triphosphate, and 0.1% NB, adjusted to a pH 7.35 with KOH. This solution contains an estimated free calcium concentration of ca. 100 nM, calculated with maxchelator (https://somapp.ucdmc.ucdavis.edu/pharmacology/bers/maxchelator/downloads.htm, program used: experimental, two chelator, two metal calculators, constants from “Chelator” program). For pharmacology, slices were preincubated for at least 10 min with ISR (3, 10, 30, and 300 nM) before recording. Recordings were performed using an EPC-10 patch-clamp amplifier (HEKA Elektronik) with a sampling rate of 20 kHz and a low-pass filter (Bessel, 5 kHz). For analysis, recordings were further digitally filtered at 1 KHz.

#### Perforated patch

Gramicidin (6 to 9 μg/ml) was used with a pipette solution containing the following: 140 mM KCl, 10 mM Hepes, 1 mM EGTA, 2 mM MgCl_2_, and 0.1% NB, adjusted to a pH 7.35 with KOH. Gramicidin containing solution was used for 2 to 3 hours. Upon seal formation, neurons were allowed to stabilize for at least 3 min before recording. Four minutes into recording, ISR (300 nM) was superfused into the bath solution. At the end of each recording, a gentle suction was applied to break into the patched membrane and to allow for sufficient cell filling with NB-containing pipette solution for >2 min.

#### Dynamic clamp

Experiments were conducted as previously described (*16*). A real-time Linux-based data acquisition program (RTXI; http://rtxi.org) was used to inject online calculated currents (acquisition rates of 10 kHz) of the NMDAR according to the following equation (*65*): *I*_NMDA_ = −*g*_NMDA_ × {1/[(1 + ([Mg]/3.57) × *e*^(−*V*m×0.062)^)]} × (*V*_m_ − *E*_NMDA_). *V*_m_ is the membrane voltage in millivolts, *E*_NMDA_ is the reversal potential for the NMDAR in millivolts, [Mg] is the external Mg^2+^ concentration in millimolars (set to 1.2 mM in this study), and *g*_NMDA_ is the conductance in nanosiemens. *g*_NMDA_ of 4, 8, 12, 16, 24, and 32 nS were applied at steady-state level for at least 1 s to generate high rates of firing activity.

The Ca_v_1.3 channel was modeled using a Hodgkin-Huxley formulation with a single activation variable (*m*) and partial inactivation via a single inactivation variable (*h*) (table S3). The model equations are given in a table below. Model parameters were fit using python to whole-cell voltage clamp recordings from lateral DA SN neurons of 1-s steps in 5-mV intervals from a −60-mV holding potential. Activation and inactivation curves were fit to peak and final currents, respectively, after eliminating passive background currents. Time constants were fit to biexponential fits of each current trace. The initial −60-mV holding potential was chosen to limit potential contamination from HCN and Ca_v_3 currents. As certain features of the voltage clamp recordings were suggestive of incomplete spatial clamp, such as delayed regenerative activation between −40 and −50 mV, simulated single-electrode voltage-clamp recordings were performed on a model containing a full morphology under simulated sodium and potassium channel blockers. These simulated experiments not only qualitatively replicated the anomalous delayed activation over the −40- to −50-mV range but also confirmed that errors introduced to recorded Ca_v_1.3 currents from imperfect space clamp are negligible at more depolarized voltages provided that Ca_v_1.3 channels are expressed proximally.

Equations for Ca_v_1.3 dynamic clamp model

**Table:** 

***G* = *G*_max_*m* [0.4 + 0.6*h*]**	
$\frac{\mathrm{dm}}{\mathrm{dt}}=\frac{m_{\text{inf}}(V)-m}{\tau_{m}(V)}$	$\frac{\mathrm{dh}}{\mathrm{dt}}=\frac{h_{\text{inf}}(V)-h}{\tau_{h}(V)}$
$m_{\text{inf}}(V)={\left[1+\text{exp}(-\frac{V+35}{5})\right]}^{-1}$	$h_{\text{inf}}(V)={\left[1+\text{exp}(\frac{V+40}{9})\right]}^{-1}$
$\tau_{m}(V)=5+5\text{exp}[-{\left(\frac{V+50}{10}\right)}^{2}]$	$\tau_{h}(V)=70+380{\left[1+\text{exp}(\frac{V+50}{5})\right]}^{-1}$

A corresponding dynamic clamp protocol for the Ca_v_1.3 model was implemented in RTXI.

### Immunohistochemistry and anatomical localization

Following in vivo recordings, animals were transcardially perfused with fixatives [4% paraformaldehyde and 0.1 M PBS (pH 7.4)]. Fixed brains were sectioned into 60-μm coronal slices using a vibrating microtome (VT1000S, Leica). Slices were rinsed in PBS and then incubated in blocking solution (0.2 M PBS with 10% horse serum, 0.5% Triton X-100, and 0.2% bovine serum albumin). Afterward, slices were incubated in carrier solution overnight with primary antibodies [polyclonal rabbit anti–tyrosine hydroxylase (TH), Millipore; 1:1000]. On the following day, slices were again washed in PBS and incubated overnight with the following secondary antibodies: goat anti-rabbit 488 (Invitrogen) and streptavidin Alexa Fluor 568 (Invitrogen). Multilabeling fluorescent immunostainings of recorded and juxtacellularly filled neurons were detected using a laser scanning microscope (Nikon Eclipse90i, Nikon GmbH). NIS-Elements C program (Nikon software) was used to acquire and export images. Overview images of the midbrain were acquired with 10× or 20× objectives, which allowed for the exact mapping of NB-labeled DA SN neurons along the SN. High-magnification images of labeled neurons were acquired with a 60× oil immersion objective. Non–NB-labeled DA SN neurons were functionally identified by their broad spike widths and slow frequency (*66*) and located by the position of the recording electrode track (fig. S10). Following in vitro recordings, slices were kept in fixatives overnight and histologically processed as described.

### Chemicals

All drugs were obtained either from Sigma-Aldrich (St. Louis, Missouri) or Tocris Bioscience (Bristol). Drugs were dissolved either in water or DMSO and stocked at −21°C before use. DMSO containing extracellular solutions did not exceed a DMSO concentration of 0.1%.

### Data analysis

Offline data analysis was performed using FITMASTER (HEKA Elektronik) and custom-written software in Igor Pro (WaveMetrics Inc.) and MATLAB. For in vitro, spike thresholds (in millivolts) were determined at *dV*_m_/*dt* > 10 mV/ms; for perforated patch-clamp recordings, a custom-written peak-detection method was applied to detect spikes. For in vivo, spikes were detected by thresholding above noise level with Igor Pro. The following *g*_NMDA_-evoked electrophysiological parameters were used for principal components analysis: *g*_NMDA_-evoked firing frequency averaged for the first three ISIs (frequency in hertz), averaged minAHP for the first four spikes (minAHP in millivolts), spike accommodation as the ratio of first ISI and second ISI (ISI1/ISI2), averaged spike threshold of the first four spikes (threshold in millivolts), and averaged spike width of the first four spikes (spike width in milliseconds). Unsupervised, hierarchical clustering (dendrogram) of the first three PCs was done agglomeratively using a Euclidian distance metric [inner squared distance (minimum variance algorithm)].

### Statistical analysis

Statistical analyses were performed using GraphPad Prism9 (GraphPad Software) and MATLAB. Initially, all datasets were tested for normality using the single-sample Kolmogorov-Smirnov test. In normally distributed datasets, statistical significance was tested using unpaired Student’s *t* test (two-tailed), paired *t* test (two-tailed), one-way analyses of variance (ANOVAs), and repeated-measures two-way ANOVAs. Post hoc tests were performed using Tukey’s, Bonferroni’s, and Dunnett’s multiple comparisons test. For datasets failing normality, Mann-Whitney test (two-tailed) or a mixed-effects model were performed. For cumulative distributions, the two-sample Kolmogorov-Smirnov test was performed to determine statistical significance. Significance was assumed against a type I error rate of 0.05 in all tests. Sample sizes, statistical tests used, and main effects for each test are separately reported in table S2.

### Ethics

All experiments and procedures involving mice were approved by the German Regierungspräsidium Darmstadt (V54-19c20/15-F40/30).

## Notes

View/request a protocol for this paper from *Bio-protocol*.
