# Supplementary material for: Cav1.3 calcium channels are full-range linear amplifiers of firing frequencies in lateral DA SN neurons
Source: Sci Adv. 2022 Jun 8;8(23):eabm4560. doi: 10.1126/sciadv.abm4560 (PMC9177074; doi:10.1126/sciadv.abm4560)
Supplement: Supplementary file 1 — Figs. S1 to S10 Tables S1 and S2 [file sciadv.abm4560_sm.pdf]

Science Advances
AAAS

Supplementary Materials for

$Ca_v1.3$ calcium channels are full-range linear amplifiers of firing frequencies in lateral DA SN neurons

Josef Shin *et al.*

Corresponding author: Jochen Roeper, roeper@em.uni-frankfurt.de

Sci. Adv. **8**, eabm4560 (2022)
DOI: 10.1126/sciadv.abm4560

This PDF file includes:

Figs. S1 to S10
Tables S1 and S2

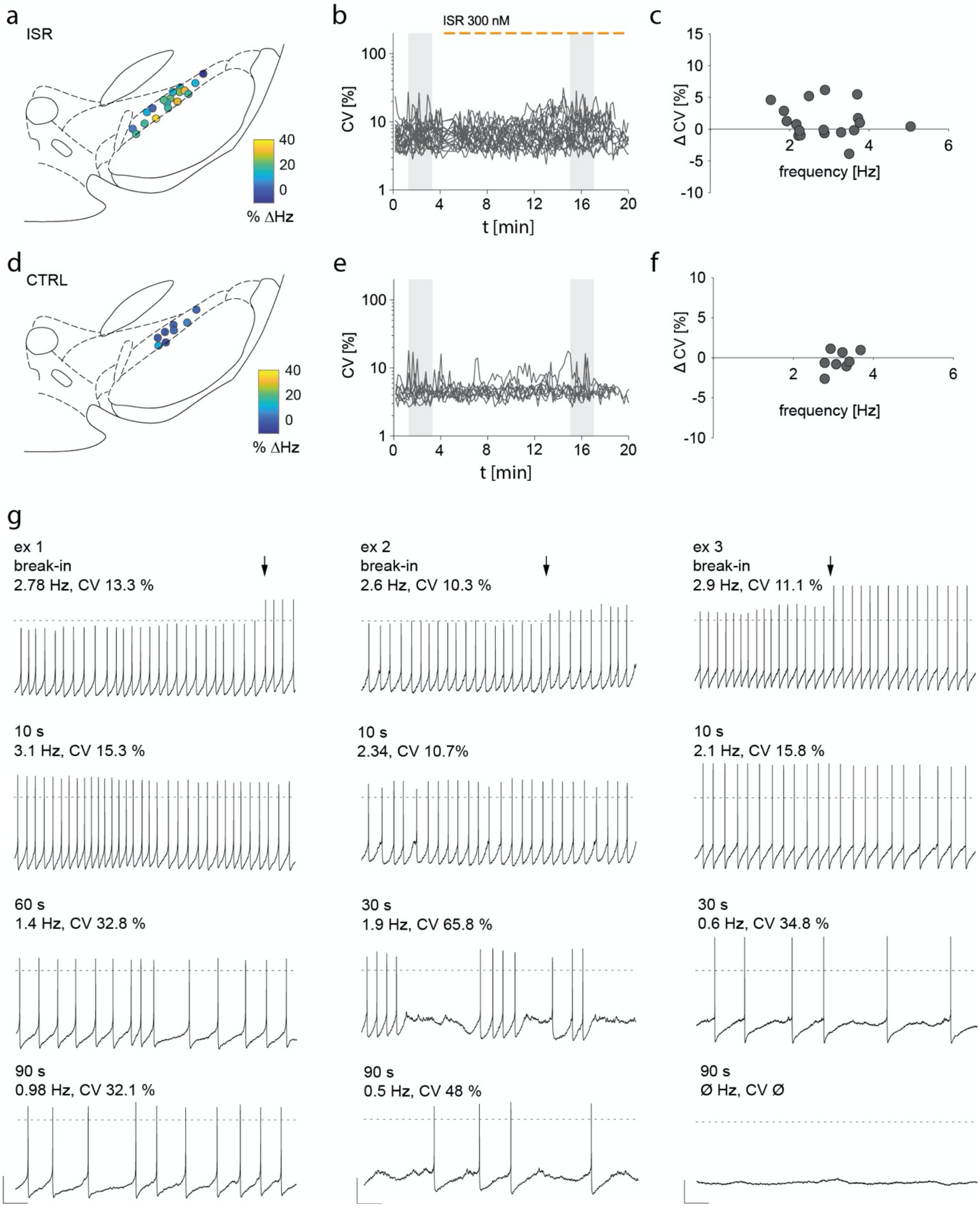

a
ISR
40
20
0
% ΔHz
b
ISR 300 nM
CV [%]
100
10
1
0 4 8 12 16 20
t [min]
c
ΔCV [%]
15
10
5
0
-5
-10
2 4 6
frequency [Hz]
d
CTRL
40
20
0
% ΔHz
e
CV [%]
t [min]
f
ΔCV [%]
frequency [Hz]
g
ex 1
break-in
2.78 Hz, CV 13.3 %
10 s
3.1 Hz, CV 15.3 %
60 s
1.4 Hz, CV 32.8 %
90 s
0.98 Hz, CV 32.1 %
ex 2
break-in
2.6 Hz, CV 10.3 %
10 s
2.34, CV 10.7%
30 s
1.9 Hz, CV 65.8 %
90 s
0.5 Hz, CV 48 %
ex 3
break-in
2.9 Hz, CV 11.1 %
10 s
2.1 Hz, CV 15.8 %
30 s
0.6 Hz, CV 34.8 %
90 s
Ø Hz, CV Ø

Fig. S1.

(a) Percentage reduction in pacemaker frequency with isradipine (300 nM) is mapped on the SN. **(b)** CV% of individual neurons plotted over recording time. Horizontal dashed line indicates superfusion of isradipine (300 nM). Shaded areas indicate range for calculation of mean CV% at baseline (minute 1 to 3) and in isradipine (minute 15 to 17). **(c)** Scatterplot of ΔCV and baseline frequency shows no correlation. **(d – f)** Data are presented as in a – c for control CV%. **(g)** Three example traces (ex. 1 – 3) displaying disruption of the perforated-patch clamp recording mode with subsequent decrease in pacemaker frequency and increase in CV% within 90 s. Dashed line indicates membrane potential at 0 mV. See Supplementary Table 2 for statistical analysis and n numbers.

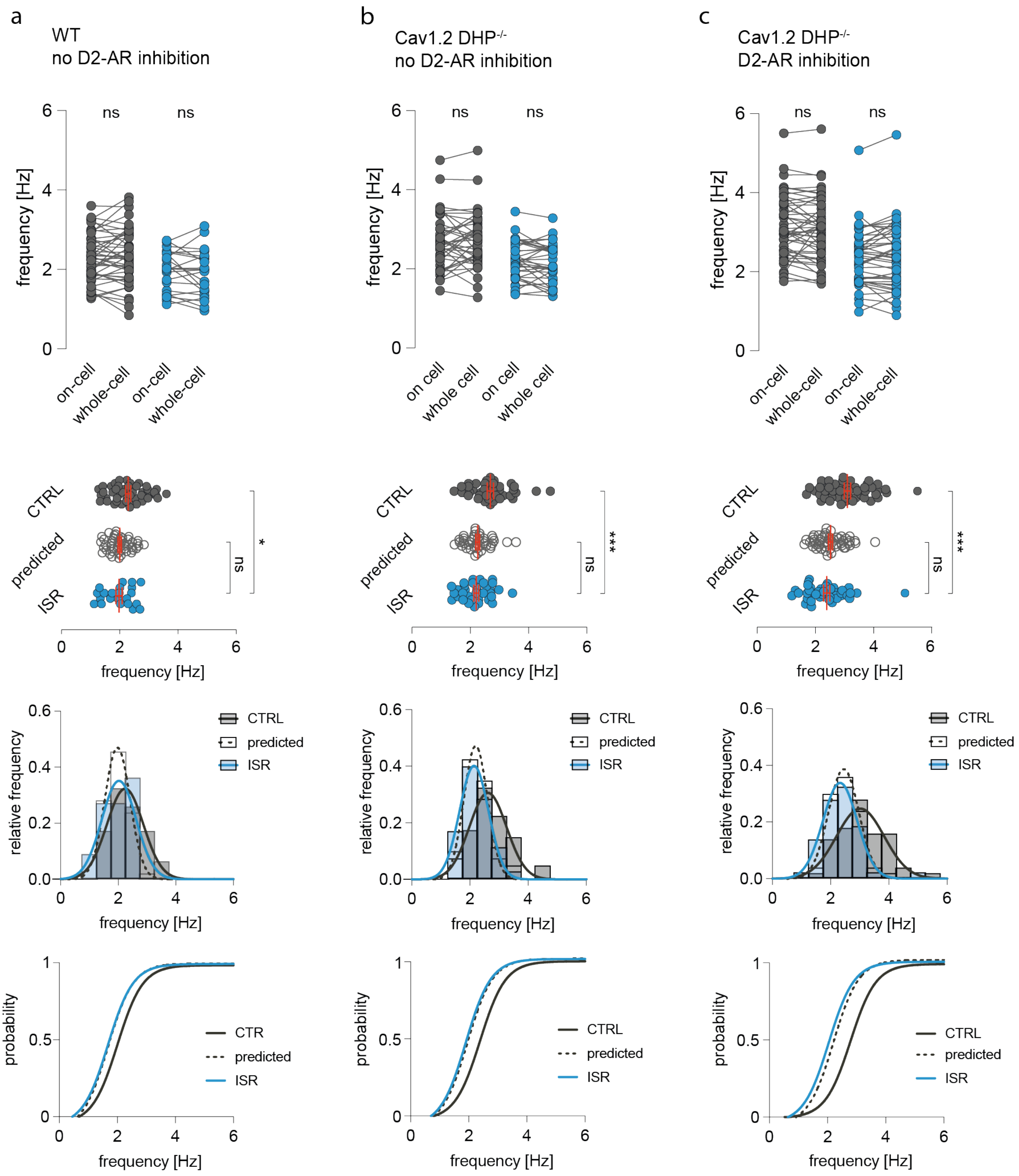

a
WT
no D2-AR inhibition
b
Cav1.2 DHP-/-
no D2-AR inhibition
c
Cav1.2 DHP-/-
D2-AR inhibition
frequency [Hz]
ns
on-cell
whole-cell
on cell
whole cell
CTRL
predicted
ISR
*
***
frequency [Hz]
relative frequency
CTRL
predicted
ISR
probability
CTR
CTRL
predicted
ISR

Fig. S2.

(a) Top row, comparison of on-cell with whole-cell pacemaker frequencies of control and isradipine – superfused DA SN neurons of WT mice. Dark circles represent control and blue circles represent recordings performed in isradipine. Bottom rows, scatterplot of control, predicted and isradipine-dependent on-cell pacemaker frequencies. Respective frequency histograms were fitted with a Gaussian function and converted to cumulative distributions. Note the very similar left-shift in the cumulative distribution for isradipine and predicted data. **(b)** Data for Cav1.2 DHP$^{-/-}$ mice are presented as in a. **(c)** Data for Cav1.2 DHP$^{-/-}$ mice under pharmacological inhibition of D2-AR are presented as in a. All data are mean ± SEM. * $P < 0.05$, *** $P < 0.001$. See Supplementary Table 2 for statistical analysis and n numbers.

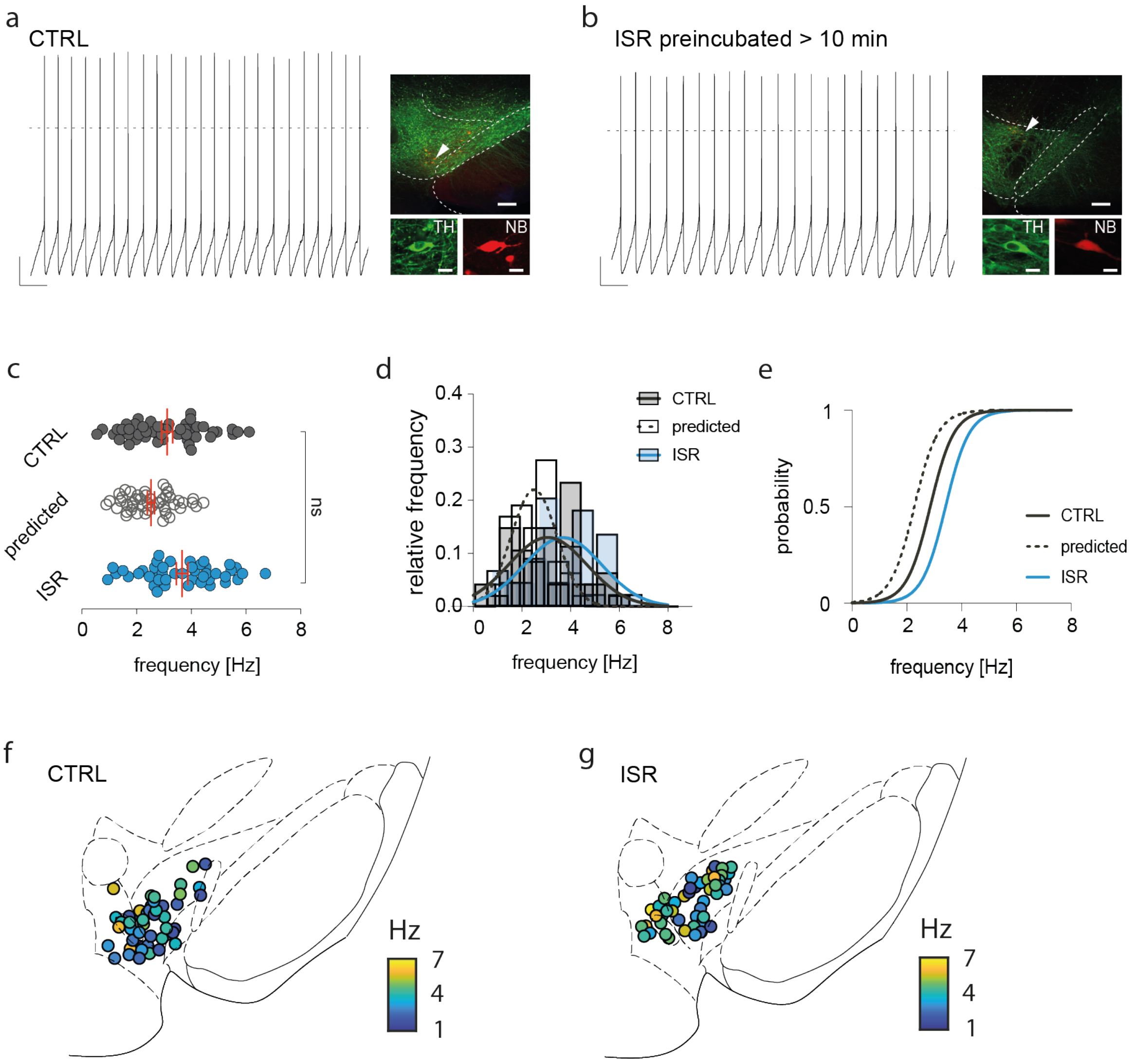
a
CTRL
TH
NB
b
ISR preincubated > 10 min
TH
NB
c
CTRL
predicted
ISR
ns
0
2
4
6
8
frequency [Hz]
d
relative frequency
0.4
0.3
0.2
0.1
0.0
CTRL
predicted
ISR
0
2
4
6
8
frequency [Hz]
e
probability
1
0.5
0
CTRL
predicted
ISR
0
2
4
6
8
frequency [Hz]
f
CTRL
Hz
7
4
1
g
ISR
Hz
7
4
1

Fig. S3.

(a) Whole-cell recording of a DA VTA neuron measured in control conditions. DA VTA neurons were labeled and histologically verified. Dashed line indicates membrane potential at 0 mV. Scale bar: 100 µm, 10 µm **(b)** DA VTA neurons were preincubation with isradipine (30 nM) for > 10 min. Dashed line indicates membrane potential at 0 mV. Scale bar: 100 µm, 10 µm **(c)** Scatter plot of control, predicted and isradipine-dependent frequencies. All experiments were performed in Cav1.2DHP$^{-/-}$ mice. **(d)** Frequency histograms of the respective distribution were fitted with a gaussian function and converted to cumulative distributions **(e)**. Pacemaker rates for each neuron in control conditions **(f)** and in isradipine **(g)** are mapped on the VTA. All data are mean ± SEM. See Supplementary Information Table 2 for statistical analysis and n numbers.

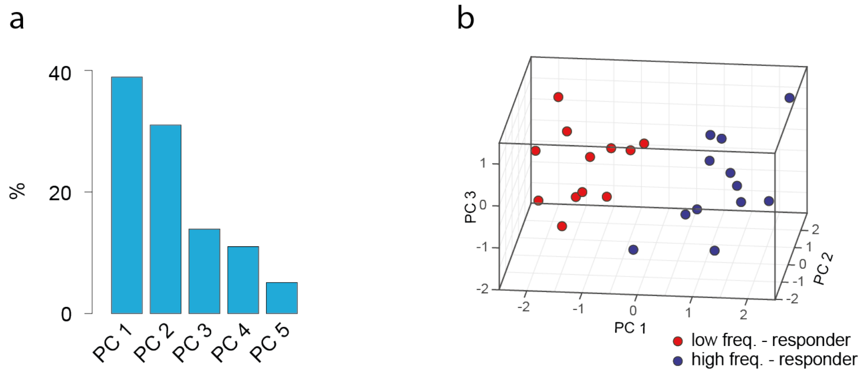

a
40
20
0
%
PC 1
PC 2
PC 3
PC 4
PC 5
b
PC 3
PC 1
PC 2
low freq. - responder
high freq. - responder

Fig. S4.

(a) Scree plot of the principal components. **(b)** Scatter plot of the first three principal components. Colors according to the hierarchical clustering of the dendrogram.

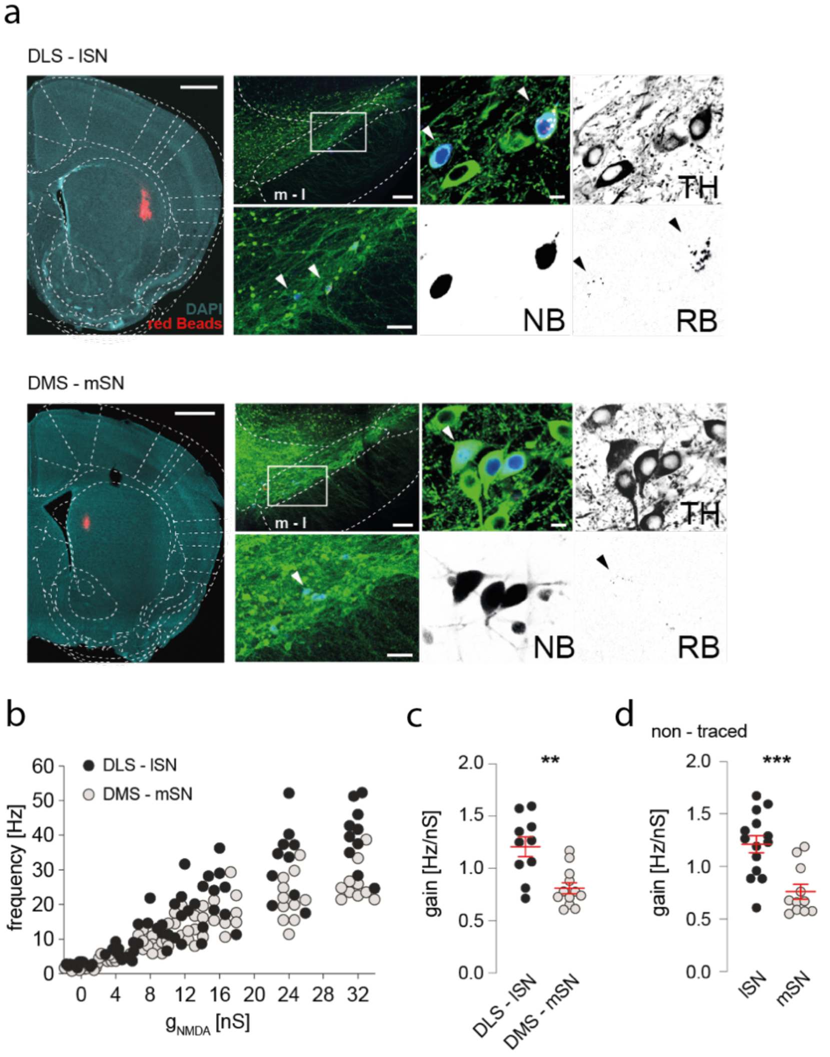

a
DLS - lSN
DAPI
red Beads
m - l
TH
NB
RB
DMS - mSN
m - l
TH
NB
RB
b
DLS - lSN
DMS - mSN
frequency [Hz]
60
50
40
30
20
10
0
0
4
8
12
16
20
24
28
32
gNMDA [nS]
c
**
gain [Hz/nS]
2.0
1.5
1.0
0.5
0.0
DLS - lSN
DMS - mSN
d
non - traced
***
gain [Hz/nS]
2.0
1.5
1.0
0.5
0.0
lSN
mSN

Fig. S5.

(a) Upper panel, injection sites for retrograde experiments in DAPI stained striatal sections using red fluorescently labeled latex beads targeted at DLS. Scale bar: 1000 µm. Retrogradely labeled DA SN neurons located in lateral SN. Arrows pointing to NB and red beads (RB) labeled TH positive lateral DA SN neurons. Scale bar: 100 µm, 50 µm, 10 µm. Lower panel, injection sites in DMS and retrogradely labeled DA SN neurons in medial SN. Scale bar: 1000 µm, 100 µm, 50 µm, 10 µm. **(b)** f-g distribution of DLS – projecting lateral and DMS – projecting medial DA SN neurons (DLS – lSN & DMS – mSN, respectively). **(c)** Higher gain (slope of individual f-g curves) in DLS – lSN neurons compared to DMS – mSN neurons. **(d)** Higher gain in lateral compared to medial DA SN (lSN & mSN, respectively) neurons. All data are mean ± SEM. ** $P < 0.01$, *** $P < 0.001$. See Supplementary Table 2 for statistical analysis and n numbers.

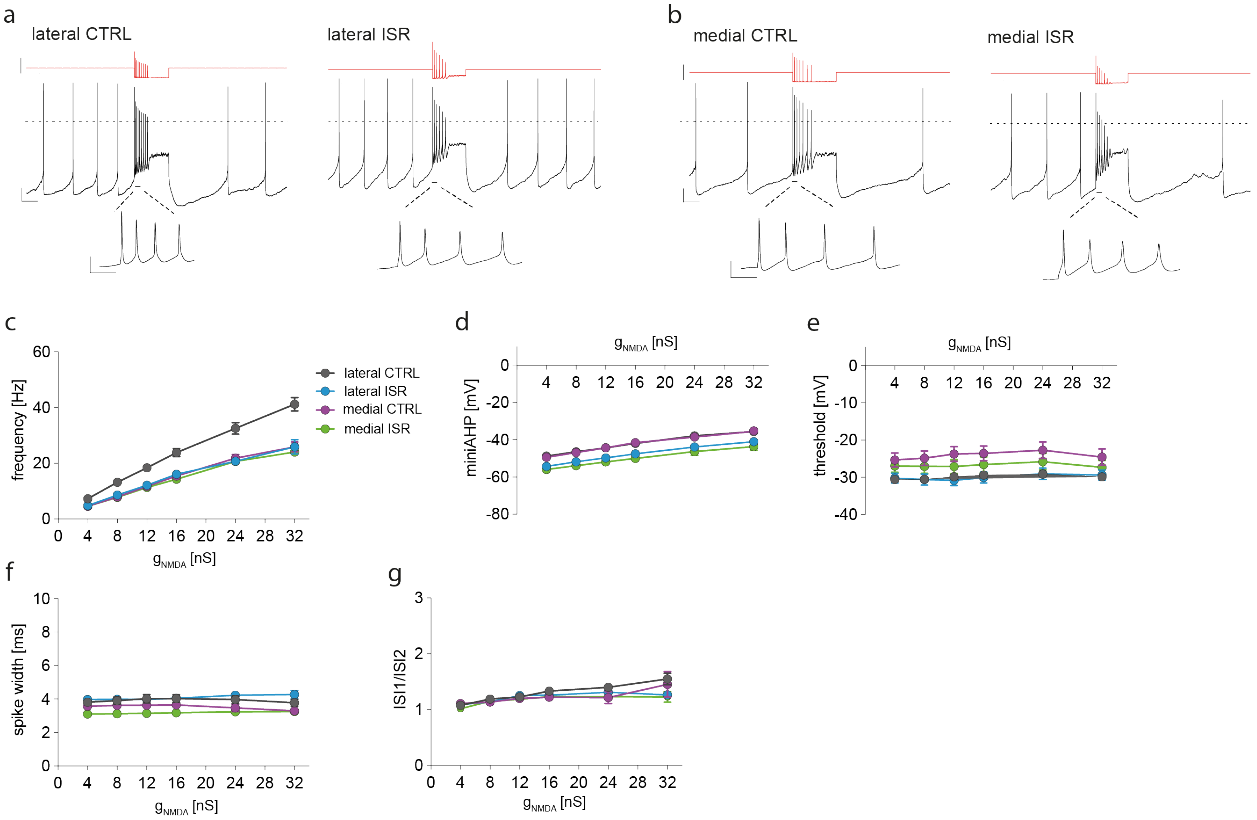
a
lateral CTRL
lateral ISR
b
medial CTRL
medial ISR
c
frequency [Hz]
lateral CTRL
lateral ISR
medial CTRL
medial ISR
d
miniAHP [mV]
e
threshold [mV]
f
spike width [ms]
g
ISI1/ISI2
g_{NMDA} [nS]

Fig. S6.

(a) g_{NMDA} was applied to expand the *in vitro* firing range in lateral DA SN neurons of WT mice in control conditions (left) or in 300 nM isradipine (right). Red line shows corresponding current injection for g_{NMDA} = 16 nS. Dashed line indicates membrane potential at 0 mV. Bottom, magnification of the first three ISIs. Scale bar: 250 pA (top), 10 mV, 500 ms (middle), 20 mV, 50 ms (bottom). **(b)** Data depicted as in **(a)** for medial DA SN neurons. Scale bar: 250 pA (top), 10 mV, 500 ms (middle), 20 mV, 50 ms (bottom). **(c)** f-g curves of lateral DA SN neurons in control (grey) or 300 nM isradipine (blue) and medial DA SN neurons in control (purple) or 300 nM isradipine (green). Note that in isradipine the firing rate of only lateral DA SN neurons was reduced to the levels of medial DA SN neurons. Afterhyperpolarisations (minAHP) **(d),** thresholds **(e)** and spike width **(f)** averaged across the first three ISIs. **(g)** Spike accommodation determined as the ratio of the first and second ISI. All data are mean ± SEM. See Supplementary Table 2 for statistical analysis and n numbers.

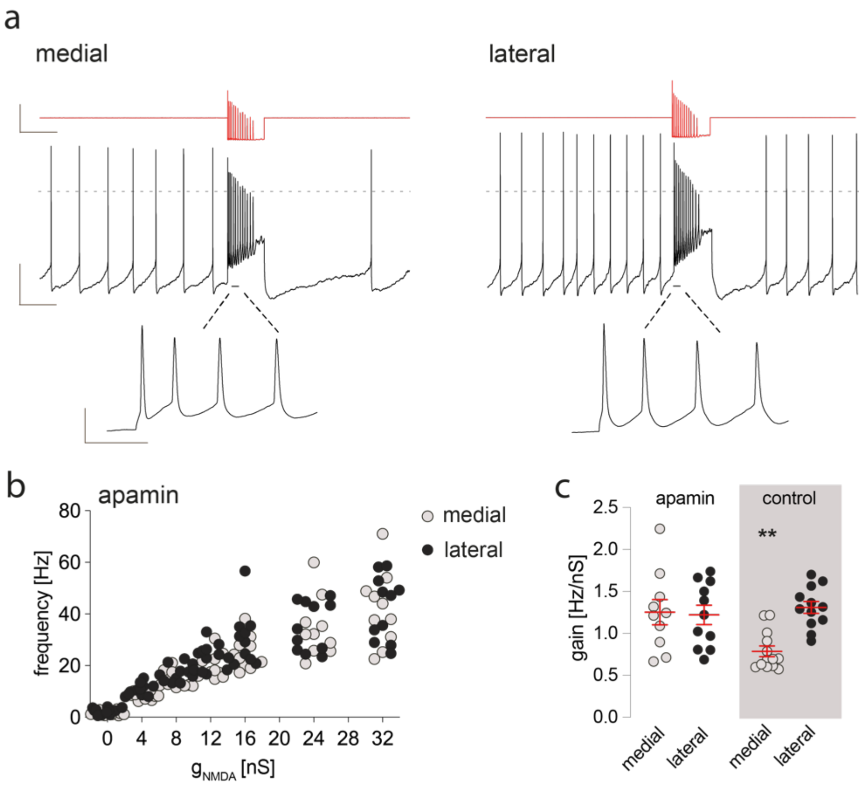

a
medial
lateral
b
apamin
80
60
40
20
0
frequency [Hz]
0
4
8
12
16
20
24
28
32
g_{NMDA} [nS]
medial
lateral
c
apamin
control
2.5
2.0
1.5
1.0
0.5
0.0
gain [Hz/nS]
**
medial
lateral
medial
lateral

Fig. S7.

(a) g_{NMDA} – induced burst responses in the presence of 300 nM apamin for medial (left) and lateral (right) DA SN neurons of WT mice. Red line shows corresponding current injection for g_{NMDA} = 16 nS. Dashed line indicates membrane potential at 0 mV. Bottom, magnification of the first three ISIs. Scale bar: 200 pA (top), 20 mV, 1 s (middle), 20 mV, 50 ms (bottom). **(b)** Scatter plot of mean frequencies averaged across the first three ISIs (f-g distribution). **(c)** Gain (slope of individual f-g curves) of medial and lateral DA SN neurons in apamin and in control conditions. Note the higher gain in medial DA SN neurons in apamin compared to control. All data are mean ± SEM. ** $P < 0.01$. See Supplementary Table 2 for statistical analysis and n numbers.

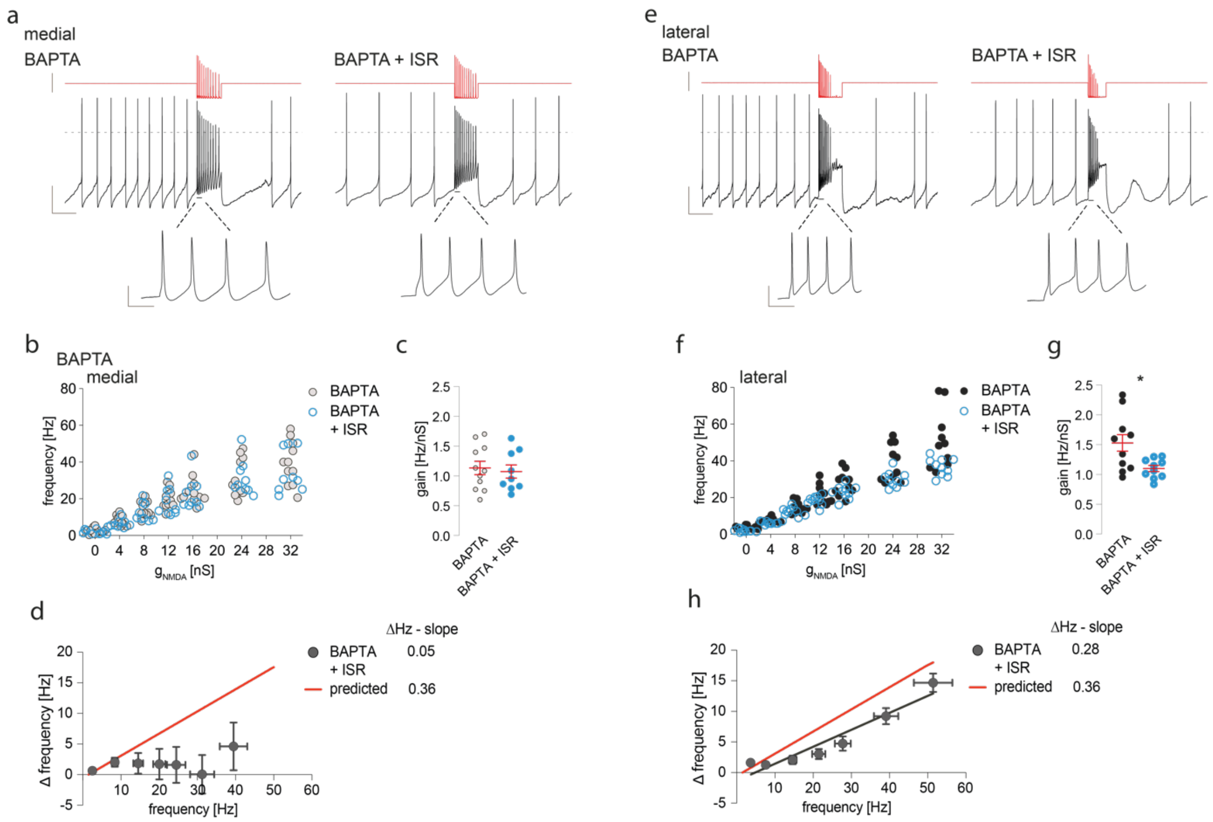

a
medial
BAPTA
BAPTA + ISR
e
lateral
BAPTA
BAPTA + ISR
b
BAPTA
medial
BAPTA
BAPTA + ISR
frequency [Hz]
g_{NMDA} [nS]
c
gain [Hz/nS]
BAPTA
BAPTA + ISR
f
lateral
g
*
d
ΔHz - slope
BAPTA + ISR 0.05
predicted 0.36
Δ frequency [Hz]
frequency [Hz]
h
ΔHz - slope
BAPTA + ISR 0.28
predicted 0.36

Fig. S8.

(a) g_{NMDA} – induced burst responses of medial DA SN neurons in Cav1.2DHP$^{-/-}$ mice with intracellular BAPTA (1 mM) in control (left) and in 30 nM isradipine (right). Red line shows corresponding current injection for g_{NMDA} = 16 nS. Dashed line indicates membrane potential at 0 mV. Bottom, magnification of the first three ISIs. Scale bar: 200 pA (top), 20 mV, 1 s (middle), 20 mV, 25 ms (bottom). **(b)** Scatter plot of mean frequencies averaged across the first three ISIs (f-g distribution) of medial DA SN neurons in BAPTA and BAPTA + isradipine. **(c)** Gain of medial DA SN neurons in BAPTA and BAPTA + isradipine. **(d)** Scatter plot of ∆frequency (= mean BAPTA – (BAPTA +ISR)) and frequency (Hz) in BAPTA. The linear regression line (red line) represent the predicted ∆frequency. **(e - h)** Data are presented as in **(a - d)** for lateral DA SN neurons. All data are mean ± SEM. * $P < 0.05$. See Supplementary Table 2 for statistical analysis and n numbers.

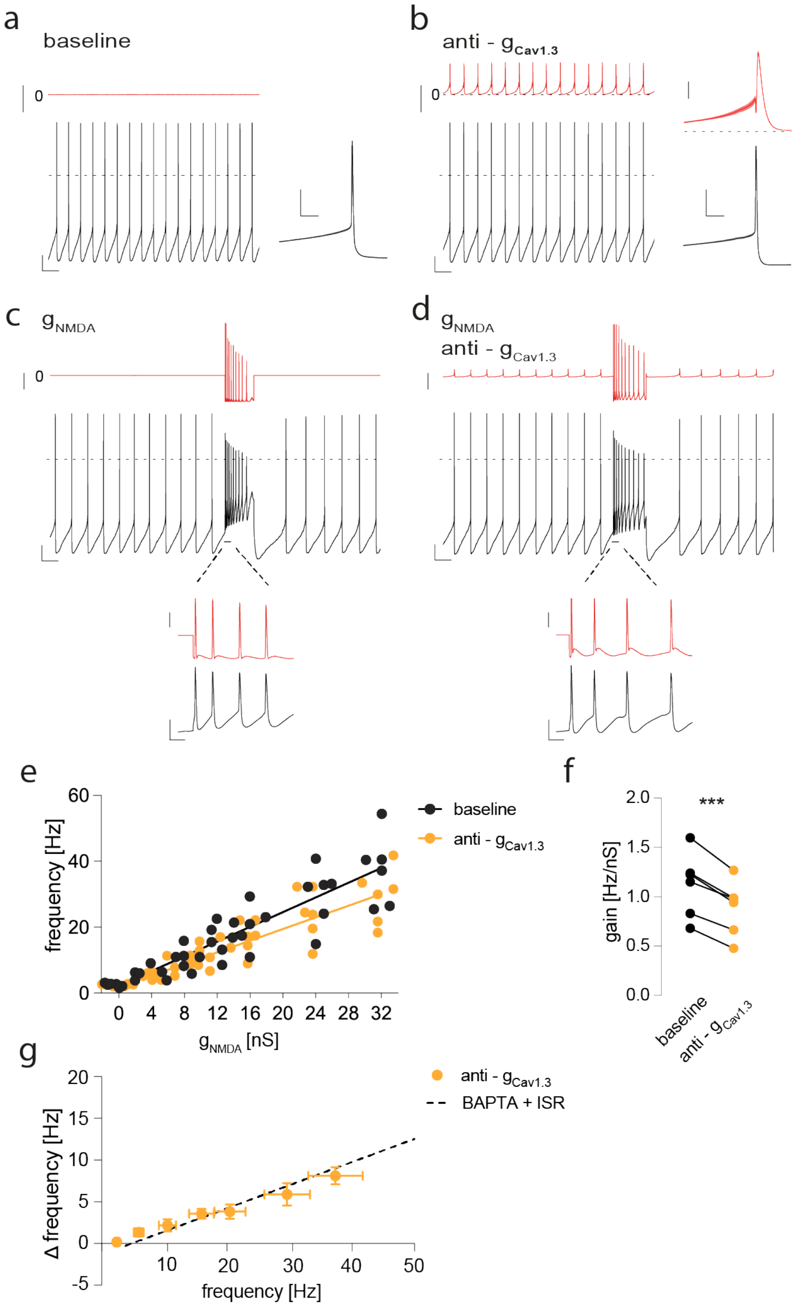
a
baseline
0
b
anti - $g_{Cav1.3}$
0
c
g_{NMDA}
0
d
g_{NMDA}
anti - $g_{Cav1.3}$
e
frequency [Hz]
60
40
20
0
0 4 8 12 16 20 24 28 32
g_{NMDA} [nS]
baseline
anti - $g_{Cav1.3}$
f
gain [Hz/nS]
2.0
1.5
1.0
0.5
0.0
***
baseline
anti - $g_{Cav1.3}$
g
Δ frequency [Hz]
20
15
10
5
0
-5
10 20 30 40 50
frequency [Hz]
anti - $g_{Cav1.3}$
BAPTA + ISR

Fig. S9.

(a) Whole-cell recording of a lateral DA SN neuron in WT mice. Red line indicates corresponding current (0 pA) for anti – $g_{Cav1.3}$ = 0 nS. Dashed line indicates membrane potential at 0 mV. Right, averaged spike waveform for spikes within a 10 s trace. Scale bar: 200 pA (top), 10 mV, 500 ms (bottom), 20 mV, 25 ms (right). **(b)** anti – $g_{Cav1.3}$ was adjusted to yield firing rates according to the linear amplification function of Cav1.3 based on baseline firing rate (red line). Dashed line indicates membrane potential at 0 mV. Right, averaged current traces (red line, shaded red area mark ± SEM) and spike waveform (black) for a 10 s trace. Scale bar: 200 pA (top, left), 10 mV, 500 ms (bottom, left), 100 pA (top, right), 20 mV, 25 ms (bottom, right). **(c)** g_{NMDA} – induced burst responses of lateral DA SN neurons. Red line shows corresponding current injection for g_{NMDA} = 16 nS. Bottom, magnification of the first three ISIs and corresponding currents (red). Scale bar: 200 pA (top), 10 mV, 500 ms (middle, whole-cell), 50 pA (middle, current), 20 mV, 25 ms (bottom). **(d)** Data are presented as in **(c)** in the presence of a priori titrated anti – $g_{Cav1.3}$. Scale bar: 200 pA (top), 10 mV, 500 ms (middle, whole-cell), 50 pA (middle, current), 20 mV, 25 ms (bottom). **(e)** Scatter plot of mean frequencies averaged across the first three ISIs (f-g distribution) of lateral DA SN neurons before (black) and after (orange) addition of anti – $g_{Cav1.3}$. **(f)** Gain before and after anti – $g_{Cav1.3}$. **(g)** Scatter plot of Δfrequency (= mean baseline – (anti – $g_{Cav1.3}$)) and baseline frequency (Hz). Compare with linear regression line of BAPTA + ISR (dashed line). All data are mean ± SEM. *** P < 0.001. See Supplementary Table 2 for statistical analysis and n numbers.

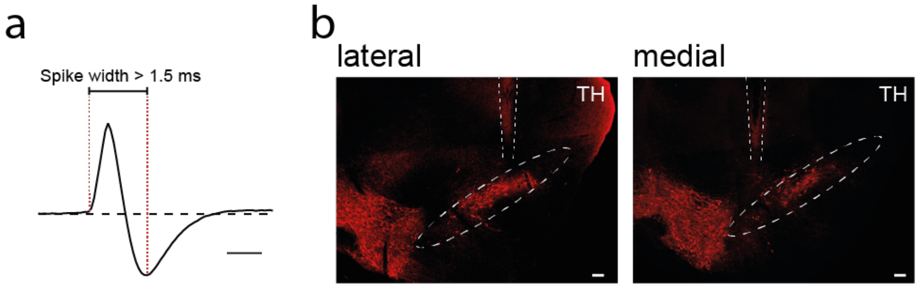
a
Spike width > 1.5 ms
b
lateral
TH
medial
TH

Fig. S10.

(a) A spike width of > 1.5 ms was used online to detect DA SN neurons. (b) The tip of the recording track points either to lateral or medial SN. Scale bar: 100 µm

Supplementary Information Table 1

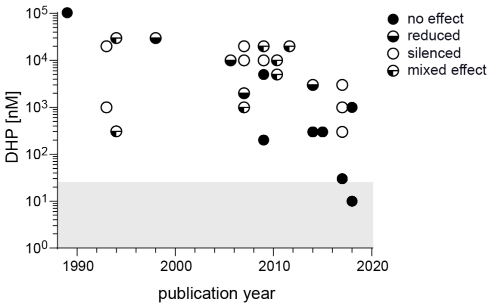

*shaded area indicate clinical range for 10 mg immediate - release isradipine (Dynacirc IR)
- peak total plasma concentration 1 ng/ml/mg Dynacirc IR (https://www.drugs.com/pro/dynacirc-cr.html)

**Published**	**Author**	**Species, age if reported**	**Recording technique**	**DHP (concentration)**	**Effect on firing activity**
1989	Fujimura & Matsuda	Guinea pig	Intracellular recording	Nifedipine (100 uM)	no effect
1993	Nedergaard et al.	Guinea pig	Intracellular recording	Nifedipine (1-20 uM)	silenced
1994	Mercuri et al.	Rats	Intracellular recording	Nifedipine (0.3 - 30 uM)	mixed
1998	Shephard & Stump	Rats	Extracellular, single-unit recording	Nifedipine (30 uM)	reduced
2007	Puopolo et al.	Mice (P 14-18)	Whole-cell patch, cell-attached	Nimodipine (1 uM)	mixed
2007	Kim et al.	Rats (P 9-14)	Whole-cell patch, cell-attached	Nimodipine (2 uM), Nifedipine (10 uM)	reduced
2007	Chan et al.	C57BL6 mice (juvenile P 21, adult > 28)	Whole-cell patch, cell-attached	Isradipine (20 uM), Nimodipine (10 uM)	silenced
2009	Guzman et al.	C57BL6 mice (P 21 -3 2)	Whole-cell patch, cell-attached	Isradipine (200 nM & 5 uM)	no effect
2009	Putzier et al.	Rats (P 14 - 21)	Whole-cell patch	Nimodipine (10 uM)	silenced
2009	Blythe et al.	Rats (P 12 - 55)	Whole-cell & perforated patch, cell-attached	Nimodipine (5 - 10 uM), Isradipine (5 - 20 uM)	mixed
2011	Drion et al.	Rats	Extracellular, single-unit recording	Nifedipine (20 uM)	mixed
2014	Dragicevic et al.	C57BL6 mice (juvenile P 13, adult P 90)	Whole-cell & perforated patch, cell-attached	Isradipine (300 nM)	no effect
2014	Branch et al.	C57BL6 mice (2-7 months & 25 - 30 months)	Whole-cell & perforated patch, loose cell-attached	Nimodipine (300 nM & 3 uM)	reduced (3 uM), no effect (300 nM)
2015	Poetschke et al.	Mice (juvenile P 13, adult P 90)	Whole-cell & perforated patch	Isradipine (300 nM)	no effect
2017	Sun et al.	C57BL6 mice (2-5 weeks old)	Whole-cell patch	Nifedipine (1 uM)	silenced
2017	Ortner et al.	C57BL6 mice (12-15 weeks old)	Perforated patch	Isradipine (30 nM, 300 nM, 3 uM)	no effect (30 nM), reduced (300 nM), silenced (3 uM)
2018	Guzman et al.	Mice (P 25-30 & P 5 - 10)	Whole-cell & perforated patch	Isradipine 10 nM & 1 uM	no effect

Table S1.

Summary of studies investigating DHP-mediated effect on firing activity of DA SN neurons. P, postnatal.

Fig. 1	Parameter	Sample size	Statistical test	Effect	p - value	Significance
c	Hz	n = 18	paired t-test (two-tailed)	t = 5.266, df = 17	p < 0.0001	p < 0.001
d	ΔHz vs Hz		single linear regression	F (1, 16) = 27.71, r2 = 0.634, slope = 0.36	p < 0.0001	p < 0.001
e	CV[%]		paired t-test (two-tailed)	t = 1.958, df = 17	p = 0.0669	n.s.
f	Hz	n = 8	paired t-test (two-tailed)	t = 0.8721, df = 7	p = 0.4121	n.s.
g	ΔHz vs Hz		single linear regression	F (1, 6) = 0.595	p = 0.469	n.s.
h	CV[%]		paired t-test (two-tailed)	t = 0.7743, df = 7	p = 0.4641	n.s.
Fig. 2						
b	Hz	CTRL n = 79, predicted n = 79, ISR n = 40	one-way ANOVA, Tukey's multiple comparisons test	F (2,195) = 7.03	p = 0.0011	p < 0.01
				CTRL vs ISR, q = 4.567, df = 195	p = 0.0041	p < 0.01
				CTRL vs predicted, q = 4.33 df = 195	p = 0.0071	p < 0.01
				ISR vs predicted, q = 1.017, df = 195	p = 0.7525	n.s.
c	Hz	CTRL n = 41, predicted n = 41, ISR n = 42	one-way ANOVA, Tukey's multiple comparisons test	F (2,121) = 8.704	p = 0.0003	p < 0.001
				CTRL vs ISR, q = 5.457, df = 121	p = 0.0005	p < 0.001
				CTRL vs predicted, q = 4.685 df = 121	p = 0.0035	p < 0.001
				ISR vs predicted, q = 0.7434, df = 121	p = 0.8589	n.s.
d	Hz	CTRL n = 50, predicted n = 50, ISR n = 43	one-way ANOVA, Tukey's multiple comparisons test	F (2,140) = 13	p < 0.0001	p< 0.001
				CTRL vs ISR, q = 6.606, df = 140	p < 0.0001	p < 0.001
				CTRL vs predicted, q = 5.711, df = 140	p = 0.0003	p < 0.001

				ISR vs predicted, q = 1.115, df = 140	p = 0.7108	n.s.
Fig. 3				F (1, 5) = 5.17		
f	ΔHz vs Hz	medial CTRL n = 11, medial ISR n = 12	single linear regression		p = 0.07	n.s.
h	ΔHz vs Hz	lateral CTRL n = 14, lateral ISR n = 15	single linear regression	F (1, 5) = 444.1, r2 = 0.989, slope = 0.357	p < 0.0001	p < 0.01
i	Frequency [Hz]	CTRL n = 14, 3 nM n = 11, 10 nM n = 10, 30 nM n = 13, 300 nM n = 15	Repeated measures two-way ANOVA, Tukey's multiple comparisons test	concentration x gNMDA, F (24, 348) = 5.219, df = 24	p < 0.0001	p < 0.01
				4 nS CTRL vs 10 nM, q = 4.006, df = 19.53	p = 0.0026	p < 0.01
				4 nS CTRL vs 30 nM, q = 5.385, df = 22.65	p< 0.0001	p < 0.01
				4 nS CTRL vs 300 nM, q = 4.824, df = 21.99	p = 0.0003	p < 0.01
				8 nS CTRL vs 10 nM, q = 3.447, df = 21.03	p = 0.0086	p < 0.01
				8 nS CTRL vs 30 nM, q = 4.499, df = 24.99	p = 0.0005	p < 0.01
				8 nS CTRL vs 300 nM, q = 5.179, df = 24.73	p < 0.0001	p < 0.01
				12 nS CTRL vs 10 nM, q = 2.912, df = 20.66	p = 0.0289	p < 0.05
				12 nS CTRL vs 30 nM, q = 3.625, df = 24.14	p = 0.0049	p < 0.01
				12 nS CTRL vs 300 nM, q = 4.775, df = 23.83	p = 0.0003	p < 0.01
				16 nS CTRL vs 10 nM, q = 2.981, df = 20.76	p = 0.0248	p < 0.05
				16 nS CTRL vs 30 nM, q = 3.816, df = 24.85	p = 0.003	p < 0.01

				16 nS CTRL vs 300 nM, q = 4.812, df = 24.63	p = 0.0003	p < 0.01
				24 nS CTRL vs 10 nM, q = 3.61, df = 24.96	p = 0.007	p < 0.01
				24 nS CTRL vs 30 nM, q = 3.855, df = 24.96	p = 0.0027	p < 0.01
				24 nS CTRL vs 300 nM, q = 4.327, df = 22.6	p = 0.001	p < 0.01
				32 nS CTRL vs 10 nM, q = 4.193, df = 20.35	p = 0.0016	p < 0.01
				32 nS CTRL vs 30 nM, q = 4.356, df = 24.73	p = 0.0008	p < 0.01
				32 nS CTRL vs 300 nM, q = 5.074, df = 24.3	p = 0.0001	p < 0.01
j	ΔHz vs Hz		single linear regression	3 nM, F (1, 5) = 96.02, r2 = 0.9505, slope 0.1344	p = 0.0002	p < 0.01
				10 nM, F (1, 5) = 135.9, r2 = 0.9645, slope 0.2811	p< 0.0001	p < 0.01
				30 nM, F (1, 5) = 394 r2 = 0.9875, slope 0.3564	p< 0.0001	p < 0.01
				300 nM, F (1, 5) = 444.1 r2 = 0.9889, slope 0.3567	p< 0.0001	p < 0.01
Fig. 4						
b	Mean frequency [Hz]	lateral n = 15	repeated measures one-way ANOVA, Dunnett's multiple comparisons test	F (4.38, 61.32) = 2.669	p = 0.036	p < 0.05
				1 min vs 20 min, q = 3.34, df = 14	p = 0.048	p< 0.05
c	fraction	lSN - CTRL ISI n = 20311, lSN - ISR ISI n = 18877	two-sample Kolmogorow-Smirnow	test statistc = 0.1181	p = 3.76 e-119	p < 0.001
d	ΔHz vs Hz		single linear regression	F (1, 13) = 4.832, r2 = 0.27, slope = 0.2657	p = 0.046	p < 0.05

Fig. 5						
b	Mean frequency [Hz]	medial n = 17	repeated measures one-way ANOVA, Dunnett's multiple comparisons test	F (3.649, 58.39) = 0.935	p = 0.4437	n.s.
c	fraction	mSN - CTRL ISI n = 26419, mSN - ISR ISI n = 26607	two-sample Kolmogorow-Smirnow	test statistc = 0.0232	p = 1.26e-6	p < 0.001
d	ΔHz vs Hz		single linear regression	F (1, 15) = 0.8428	p = 0.3731	n.s.
Suppl. Fig. 1						
c	ΔCV vs Hz		single linear regression	F(1,16) = 0.4411	p = 0.516	n.s.
f	ΔCV vs Hz		single linear regression	F(1,6) = 1.572	p = 0.2565	n.s.
Suppl. Fig. 2						
a	Hz on-cell vs whole-cell	CTRL n = 46	paired t-test (two-tailed)	t = 0.793, df = 45	p = 0.4319	n.s.
		ISR n = 22	paired t-test (two-tailed)	t = 1.132, df = 21	p = 0.2703	n.s.
	Hz	CTRL n = 46, predicted n = 46, ISR n = 22	one-way ANOVA, Tukey's multiple comparisons test	F (2,111) = 4.873	p = 0.0094	p < 0.01
				CTRL vs ISR, q = 3.402, df = 111	p = 0.0465	p < 0.05
				CTRL vs predicted, q = 3.944, df = 111	p = 0.017	p < 0.05
				ISR vs predicted, q = 0.2291, df = 111	p = 0.9856	n.s.
b	Hz on-cell vs whole-cell	CTRL n = 40	paired t-test (two-tailed)	t = 0.2763, df = 39	p = 0.7838	n.s.
		ISR n = 35	paired t-test (two-tailed)	t = 1.504, df = 34	p = 0.1418	n.s.

	Hz	CTRL n = 40, predicted n = 40, ISR n = 35	one-way ANOVA, Tukey's multiple comparisons test	F (2,112) = 9.01	p = 0.0002	p< 0.001
				CTRL vs ISR, q = 5.356, df = 112	p = 0.0007	p < 0.001
				CTRL vs predicted, q = 4.962, df = 112	p = 0.0019	p < 0.001
				ISR vs predicted, q = 0.5623, df = 112	p = 0.9166	n.s.
c	Hz on-cell vs whole-cell	CTRL n = 50	paired t-test (two-tailed)	t = 0.7843, df = 49	p = 0.4366	n.s.
		ISR n = 43	paired t-test (two-tailed)	t = 0.7652, df = 43	p = 0.4484	n.s.
	Hz	CTRL n = 50, predicted n = 50, ISR n = 43	one-way ANOVA, Tukey's multiple comparisons test	F (2,140) = 15.36	p < 0.0001	p< 0.001
				CTRL vs ISR, q = 7.229, df = 140	p < 0.0001	p < 0.001
				CTRL vs predicted, q = 6.132, df = 140	p < 0.0001	p < 0.001
				ISR vs predicted, q = 1.333, df = 140	p = 0.6146	n.s.
Suppl. Fig. 3						
c	frequency [Hz]	CTRL n = 47, predicted n = 47, ISR n = 44	one-way ANOVA, Tukey's multiple comparisons test	F (2, 135) = 9.582	p = 0.0001	p < 0.001
				CTRL vs ISR, q = 2.974, df = 135	p = 0.093	n.s.
				CTRL vs predicted, q = 3.266, df = 135	p = 0.058	n.s.
				ISR vs predicted, q = 6.186, df = 135	p < 0.0001	p < 0.001
Suppl. Fig. 5						
c	gain [Hz/nS]	DLS - lSN n = 10, DMS - mSN n = 12	unpaired t-test (two-tailed)	t = 3.84, df = 20	p = 0.001	p < 0.01
d	gain [Hz/nS]	lSN n = 14, mSN n = 11	unpaired t-test (two-tailed)	t = 4.084, df = 23	p = 00005	p < 0.001

Suppl. Fig. 6						
c	frequency [Hz]	lateral CTRL n = 14, lateral ISR n = 15, medial CTRL n = 11, medial ISR n = 12	Repeated measures two-way ANOVA, Bonferroni's multiple comparisons test	population x gNMDA, F (15,235) = 9.489, df = 15	p < 0.0001	p < 0.001
				4 nS medialCTRL vs lateralCTRL, t = 4.16, df = 22.68	p = 0.0012	p < 0.01
				4 nS medialCTRL vs lateral ISR, t = 0.53, df = 21.83	p > 0.9999	n.s.
				8 nS medialCTRL vs lateralCTRL, t = 4.691, df = 21.64	p = 0.0003	p < 0.001
				8 nS medialCTRL vs lateralISR, t = 0.8481, df = 23.96	p > 0.9999	n.s.
				12 nS medialCTRL vs lateralCTRL, t = 4.691, df = 21.3	p = 0.0004	p < 0.001
				12 nS medialCTRL vs lateralISR, t = 0.3435, df = 23.88	p > 0.9999	n.s.
				16 nS medialCTRL vs lateralCTRL, t = 4.621, df = 21.63	p = 0.0004	p < 0.001
				16 nS medialCTRL vs lateralISR, t = 0.4631, df = 23.94	p > 0.9999	n.s.
				24 nS medialCTRL vs lateralCTRL, t = 4.96, df = 22.17	p = 0.0002	p < 0.001
				24 nS medialCTRL vs lateralISR, t = 0.622, df = 23.98	p > 0.9999	n.s.
				32 nS medialCTRL vs lateralCTRL, t = 4.325, df = 22.17	p = 0.0008	p < 0.001
				32 nS medialCTRL vs lateralISR, t = 0.009, df = 18.81	p > 0.9999	n.s.

d	minAHP [mV]		Repeated measures two-way ANOVA, Bonferroni's multiple comparisons test	population effect F (3, 41) = 8.891, df = 3	p = 0.0001	p < 0.001
				4 nS medialCTRL vs lateralCTRL, t = 0.2299, df = 19.76	p > 0.9999	n.s.
				4 nS medialCTRL vs lateral ISR, t = 4.323, df = 19.76	p = 0.0011	p < 0.01
				8 nS medialCTRL vs lateralCTRL, t = 0.1017 df = 19.83	p > 0.9999	n.s.
				8 nS medialCTRL vs lateralISR, t = 4, df = 16.69	p = 0.0029	p < 0.01
				12 nS medialCTRL vs lateralCTRL, t = 0.1826, df = 19.98	p > 0.9999	n.s.
				12 nS medialCTRL vs lateralISR, t = 4.067, df = 19.4	p = 0.0019	p < 0.01
				16 nS medialCTRL vs lateralCTRL, t = 0.367, df = 19.79	p > 0.9999	n.s.
				16 nS medialCTRL vs lateralISR, t = 3.875, df = 18.06	p = 0.0033	p < 0.01
				24 nS medialCTRL vs lateralCTRL, t = 0.175, df = 19.87	p > 0.9999	n.s.
				24 nS medialCTRL vs lateralISR, t = 3.11, df = 19.34	p = 0.0169	p < 0.05
				32 nS medialCTRL vs lateralCTRL, t = 0.1788, df = 18.77	p > 0.9999	n.s.
				32 nS medialCTRL vs lateralISR, t = 2.762, df = 16.42	p = 0.04	p < 0.05

e	threshold [mV]		Repeated measures two-way ANOVA, Bonferroni's multiple comparisons test	population effect F (3, 46) = 3.597, df = 3	p = 0.02	p < 0.05
				4 nS medialCTRL vs lateralCTRL, t = 2.7, df = 11.62	p = 0.059	n.s.
				4 nS medialCTRL vs lateral ISR, t = 2.11, df = 18.99	p = 0.1453	n.s.
				8 nS medialCTRL vs lateralCTRL, t = 2.811 df = 11.22	p = 0.499	p < 0.05
				8 nS medialCTRL vs lateralISR, t = 2.34, df = 19.75	p = 0.089	n.s.
				12 nS medialCTRL vs lateralCTRL, t = 2.969, df = 11.77	p = 0.0358	p < 0.05
				12 nS medialCTRL vs lateralISR, t = 2.922, df = 18.94	p = 0.0263	p < 0.05
				16 nS medialCTRL vs lateralCTRL, t = 2.737, df = 12.72	p = 0.0517	n.s.
				16 nS medialCTRL vs lateralISR, t = 2.614, df = 19.55	p = 0.0504	n.s.
				24 nS medialCTRL vs lateralCTRL, t = 2,663, df = 13.79	p = 0.0562	n.s.
				24 nS medialCTRL vs lateralISR, t = 2.352, df = 13.79	p = 0.0169	p < 0.05
				32 nS medialCTRL vs lateralCTRL, t = 0.1788, df = 18.77	p = 0.089	n.s.
				32 nS medialCTRL vs lateralISR, t = 1,877, df = 17.3	p = 0.2323	n.s.

f	spike width [ms]		Repeated measures two-way ANOVA, Bonferroni's multiple comparisons test	population x gNMDA, F (15, 205) = 5.327, df = 15	p < 0.0001	p < 0.001
				4 nS medialCTRL vs lateralCTRL, t = 0,9769, df = 18.49	p = 0.059	n.s.
				4 nS medialCTRL vs lateral ISR, t = 1,778, df = 16.18	p = 0.1453	n.s.
				8 nS medialCTRL vs lateralCTRL, t = 1,028 df = 19.21	p = 0.499	p < 0.05
				8 nS medialCTRL vs lateralISR, t = 1,500, df = 15.99	p = 0.089	n.s.
				12 nS medialCTRL vs lateralCTRL, t = 1,262, df = 19.95	p = 0.0358	p < 0.05
				12 nS medialCTRL vs lateralISR, t = 1,446, df = 15.19	p = 0.0263	p < 0.05
				16 nS medialCTRL vs lateralCTRL, t = 1,379, df = 20	p = 0.0517	n.s.
				16 nS medialCTRL vs lateralISR, t = 1,797, df = 17.25	p = 0.0504	n.s.
				24 nS medialCTRL vs lateralCTRL, t = 1,616, df = 18,36	p = 0.0562	n.s.
				24 nS medialCTRL vs lateralISR, t = 2,670, df = 16.06	p = 0.0169	p < 0.05
				32 nS medialCTRL vs lateralCTRL, t = 1,740, df = 18.93	p = 0.089	n.s.
				32 nS medialCTRL vs lateralCTRL, t = 3,275, df = 19.51	p = 0.089	n.s.

g	ISI1/ISI2		Mixed-effects model, Bonferroni's multiple comparisons test	population x gNMDA, F (15, 221) = 1.61	p = 0.0724	n.s.
Suppl. Fig. 7						
c	gain [Hz/nS]	apamin medial n = 10, apamin lateral n = 11	one-way ANOVA, Tukey's multiple comparisons test	F (3, 42) = 6.679	p = 0.0009	p < 0.001
				apamin medial vs ctrl media, q = 4.898, df = 42	p = 0.0066	p < 0.01
				apamin lateral vs ctrl lateral, q = 0.6457, df = 42	p = 0.9680	n.s.
Suppl. Fig. 8						
c	gain [Hz/nS]	BAPTA n = 9, BAPTA + ISR n = 11	unpaired t-test (two-tailed)	t = 0.378, df = 18	p = 0.75	n.s.
d	ΔHz vs Hz		single linear regression		p = 0.298	n.s.
g	gain [Hz/nS]	BAPTA n = 11, BAPTA + ISR n = 10	unpaired t-test (two-tailed)	t = 2.792, df =. 19	p = 0.011	p < 0.05
h	ΔHz vs Hz		single linear regression	F (1,5) = 53.44, r2 = 0.91, slope = 0.2773	p = 0.0008	p < 0.001
Suppl. Fig. 9						
f	gain [Hz/nS]	n = 6	paired t-test (two-tailed)	t = 8.668, df = 5	p = 0.0003	p < 0.001

Table S2.
statistical analyses
